# Digital Approaches to Automated and Machine Learning Assessments of Hearing: Scoping Review

**DOI:** 10.2196/32581

**Published:** 2022-02-02

**Authors:** Jan-Willem Wasmann, Leontien Pragt, Robert Eikelboom, De Wet Swanepoel

**Affiliations:** 1 Department of Otorhinolaryngology, Donders Institute for Brain, Cognition and Behaviour Radboud University Medical Centre Nijmegen Netherlands; 2 Ear Science Institute Australia Subiaco Australia; 3 Ear Sciences Centre Medical School The University of Western Australia Perth Australia; 4 Department of Speech-Language Pathology and Audiology University of Pretoria Pretoria South Africa

**Keywords:** audiology, automated audiometry, automatic audiometry, automation, digital health technologies, digital hearing health care, machine learning, remote care, self-administered audiometry, self-assessment audiometry, user-operated audiometry, digital health, hearing loss, digital hearing, digital devices, mobile phone, telehealth

## Abstract

**Background:**

Hearing loss affects 1 in 5 people worldwide and is estimated to affect 1 in 4 by 2050. Treatment relies on the accurate diagnosis of hearing loss; however, this first step is out of reach for >80% of those affected. Increasingly automated approaches are being developed for self-administered digital hearing assessments without the direct involvement of professionals.

**Objective:**

This study aims to provide an overview of digital approaches in automated and machine learning assessments of hearing using pure-tone audiometry and to focus on the aspects related to accuracy, reliability, and time efficiency. This review is an extension of a 2013 systematic review.

**Methods:**

A search across the electronic databases of PubMed, IEEE, and Web of Science was conducted to identify relevant reports from the peer-reviewed literature. Key information about each report’s scope and details was collected to assess the commonalities among the approaches.

**Results:**

A total of 56 reports from 2012 to June 2021 were included. From this selection, 27 unique automated approaches were identified. Machine learning approaches require fewer trials than conventional threshold-seeking approaches, and personal digital devices make assessments more affordable and accessible. Validity can be enhanced using digital technologies for quality surveillance, including noise monitoring and detecting inconclusive results.

**Conclusions:**

In the past 10 years, an increasing number of automated approaches have reported similar accuracy, reliability, and time efficiency as manual hearing assessments. New developments, including machine learning approaches, offer features, versatility, and cost-effectiveness beyond manual audiometry. Used within identified limitations, automated assessments using digital devices can support task-shifting, self-care, telehealth, and clinical care pathways.

## Introduction

### Background

Hearing loss affects 1.5 billion persons worldwide and is expected to increase by another billion by 2050 [1,2]. Hearing testing is the first step toward appropriate and timely treatment. Unfortunately, most persons affected with hearing loss are unable to access hearing assessments, with less than one hearing health professional for every million people in regions such as Africa [2,3]. Increasingly automated approaches (all aspects of the method associated with automated audiometry), including machine learning, are being developed and made available to provide self-administered hearing assessments. The term *automated audiometry* refers to all hearing tests that are self-administered from the point the test starts. More specifically, in this review, we define automated audiometry as calibrated pure-tone threshold audiometry in any setting (ie, hearing health care, occupational health, and community settings) that is self-administered from the point the test starts. Machine learning refers to model-based approaches that learn from examples (data) instead of being programmed with rules [4]. As the direct involvement of professionals is not required, automated approaches enable health care pathways with the potential to increase accessibility, efficiency, and scalability. Digital (health) technologies, including apps, smartphones, tablets, and wearables, can acquire data remotely; expand the reach and precision of clinicians; and facilitate more personalized hearing health care within a network of distributed expertise [5,6]. Recent examples of automated hearing assessments include clinical grade and consumer-grade applications [7]. General global health trends suggest that increased availability of diagnostic tools could lower health care costs and improve quality of life [8]. For example, in Parkinson disease, remote care based on wearables provides ecologically valid methods for monitoring and evaluating symptoms [9,10]. In tuberculosis screening in low-resource settings, an automated diagnosis can increase the sensitivity of identifying persons at risk while reducing costs [11]. Self-assessment using eHealth vision tools improves access to diagnosis and facilitates timely diagnosis, although consistent criteria for referring to the clinical pathway and validity and reliability of eHealth tools are still a concern [12].

Timely detection and treatment of hearing loss are essential to enable optimal outcomes and quality of life across the life span [2]. Untreated hearing loss restricts language development and educational potential in children and is associated with a more rapid cognitive decline in adults [13]. It may lead to social isolation, lower socioeconomic status, increased social disparities, and decreased health, resulting in lower quality of life at the individual level and substantial costs at the community level [14,15]. Importantly, treating hearing loss in midlife has been identified as the largest potentially modifiable risk factor for developing dementia in later life [16]. The global annual cost of untreated hearing loss is US $980 million [14]. Global health investment models indicate a significant return on investment in both hearing diagnosis and treatment [2]. The capacity of the entire clinical pathway should be increased as a bottleneck looms if the accessibility of diagnosis is increased faster than the availability of affordable treatment and rehabilitation.

Automated self-test options are important for detecting and diagnosing hearing loss to direct timely and appropriate treatments. The overwhelming majority of treatments are for permanent age-related and noise-induced hearing loss; however, a significant portion of the population requires medical treatment for hearing loss [1]. The onset of the COVID-19 pandemic has further emphasized the importance of self-testing approaches [17,18]. Automation on digital devices is a powerful enabler of alternative diagnostic pathways that can include home-based testing, low-touch service models outside traditional clinic settings, and decentralized community-based models that rely on task shifting to minimally trained facilitators [19].

Automation in hearing assessment is not a new concept and dates back to >7 decades [20]. In recent years, it has resurged with the convergence of digital technologies and machine learning approaches. The primary tool for hearing assessment is pure-tone audiometry, which describes the degree of hearing loss relative to normal hearing, expressed in decibels hearing level across specific frequencies (125-8000 Hz). Pure-tone audiometry can also differentiate the type of hearing loss, that is, sensorineural or conductive, when bone conduction and air conduction transducers are used. Machine learning–based threshold-seeking approaches, known as Bayesian active learning, have demonstrated their potential to optimize efficiency and increase the precision of automated hearing assessments [21]. The increased efficiency comes from the ability of these methods to target trials to those areas of the frequency space where the estimation has the greatest uncertainty [22,23].

### Objective

In 2013, a systematic review that included 29 reports on automated audiometry showed that automated procedures have comparable accuracy with that of manual procedures when performing air conduction audiometry. Although a few validated automated procedures that included automated bone conduction audiometry had been reported, machine learning–based audiometry approaches had not been reported yet, and approaches were rarely validated in children or hard-to-test populations [24]. Since 2013, there has been significant work and innovation in this area, which calls for an update and extension of the previous review. This study aims to provide the current status of automation and machine learning approaches in hearing assessment using validated pure-tone audiometry with potential indicators of accuracy, reliability, and efficiency of these approaches.

## Methods

We conducted a systematic scoping review of the peer-reviewed literature on automated and machine learning approaches to validate pure-tone threshold audiometry using digital technologies by considering accuracy, reliability, and efficiency. This review followed the methodological framework outlined by Arksey and O’Malley [25].

### Identifying Potentially Relevant Records

A search across the electronic databases of PubMed, IEEE, and Web of Science was conducted to identify relevant reports from the peer-reviewed literature. Complementary and redundant search terms were applied to ensure thorough coverage and cross-checking of the search findings. In the PubMed database, medical subject headings and relevant keywords were collected to determine all records related to the study aim. The following synonyms of, and closely related terms to, automated audiometry were used: automatic audiometry, self-administered audiometry, self-assessment audiometry, and user-operated audiometry. The complete set of terms and the applied search strategy are provided in Multimedia Appendix 1. The IEEE database is engineering oriented, and only relevant keywords based on audiometry were used, as it was assumed that any result in audiometry would be highly associated with automated audiometry. The Web of Science database is known to index the PubMed and IEEE databases and was explored using search terms similar to the PubMed search. After preliminary explorations to identify appropriate keywords, we conducted a search on July 8, 2020, and updated it on January 12, 2021, and July 6, 2021. The search included all reports that met the inclusion criteria published from January 1, 2012, to June 30, 2021. The start date was chosen as we regard this scoping review as an extension and generalization of a previous (systematic) review by Mahomed et al [24], which included studies up to July 20, 2012.

### Selecting Relevant Records

Reports had to meet the following three inclusion criteria: (1) the report had to be about automated or machine learning and pure-tone frequency-specific threshold audiometry, (2) it had to be written in English, and (3) the automated threshold audiometry had to be compared against the gold standard or reasonable standard. The gold standard is defined as manual audiometry in a sound booth according to the International Organization for Standardization standards. Automated audiometry also needed to be performed inside a sound booth, and the results needed to be compared with the gold standard. A reasonable standard for validation was defined as either a within-subject comparison between the gold standard and the automated audiometry in an unconventional setting (eg, a quiet room) or a within-subject comparison between a validated automated audiometry approach and an experimental approach of audiometry in the same unconventional setting.

We excluded reports on screening audiometry (eg, provided pass or refer as an outcome) rather than threshold audiometry, review papers, and studies reporting approaches that were not compared with the gold or reasonable reference standard.

The first phase of screening was based on the title. If the title indicated that content was within the scope of the research question (ie, automated or machine learning approaches in diagnostic hearing assessment), the report was included in the second screening phase. In the second phase, the abstracts of the remaining reports were assessed using the inclusion and exclusion criteria stated earlier.

**Figure 1:**
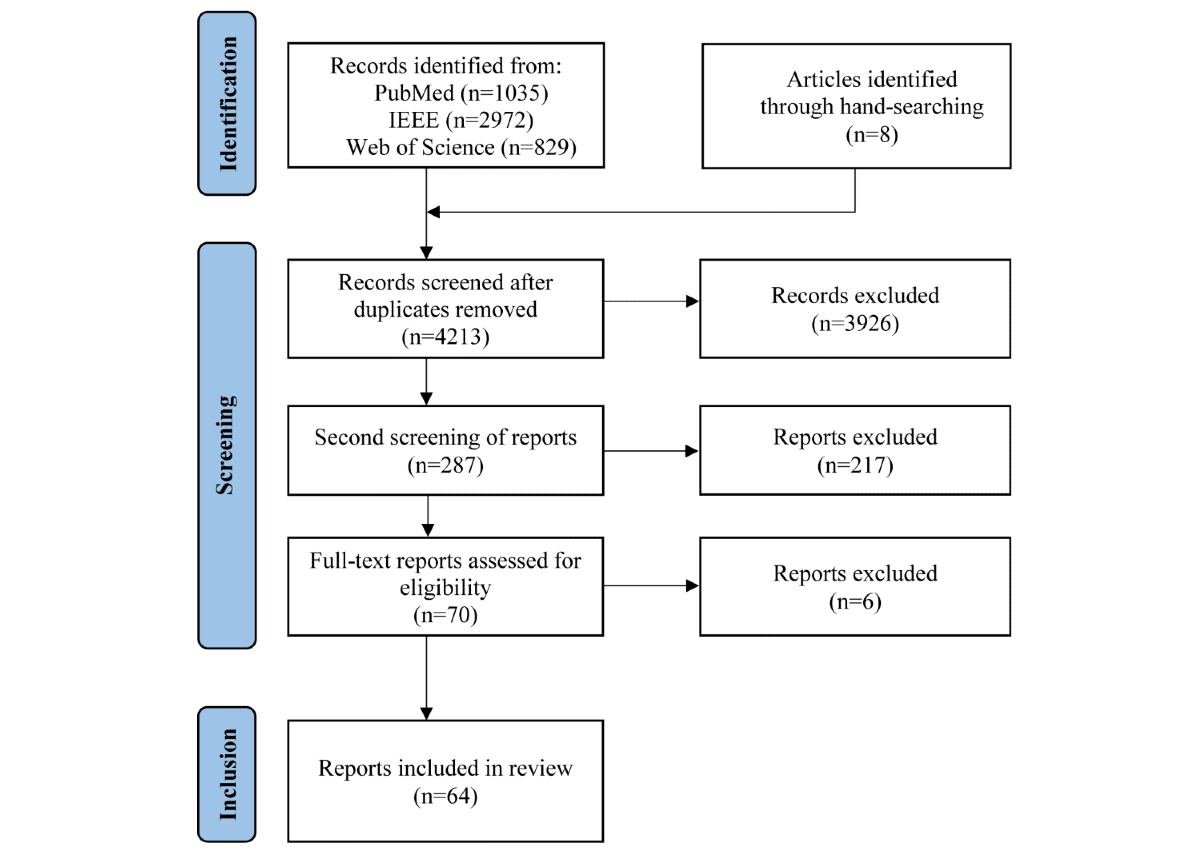
PRISMA (Preferred Reporting Items for Systematic Reviews and Meta-Analyses) flow diagram of the screening process.

Two researchers (LP and JWW) conducted the abstract screening. They were blinded from each other to avoid confirmation bias. After the screening, the researchers discussed any disagreements to reach an agreement. When in doubt, the report was admitted to the third, full-text review phase. In this phase, all the remaining reports were reviewed in full to determine whether the inclusion criteria were met. As can be seen in the PRISMA (Preferred Reporting Items for Systematic Reviews and Meta-Analyses) flow diagram (Figure 1), the resulting selection of reports was complemented by additional reports. After some reports were clustered as having identical approaches (explained in *Collating Approaches, Summarizing, and Reporting the Results*), additional reports were added to avoid missing validation data of these clustered approaches. These additional reports were published before the inclusion date criteria (from before January 1, 2012) or did not appear in the search and were added based on the reference lists of the already included reports.

### Extracting Data Items

A template for grading the reports was agreed upon by all the authors (Multimedia Appendix 2 [26]). Two researchers (LP and JWW) independently extracted information directly relevant to the scoping review question. In cases of disagreement, a consensus was reached after discussion between the 2 researchers. The compulsory data fields were test frequency and intensity range; response method; test equipment, including the type of transducers; calibration; hardware; test quality control; accuracy; reliability; efficiency; validation; and test population. In the report by Mahomed et al [24], the accuracy and reliability of manual and automated approaches demonstrated equivalent performances. Time efficiency had primarily been reported by comparing the testing times of manual and automated audiometry [27-29]. The reports on machine learning audiometry explicitly used the number of trials or stimuli needed to converge to a certain precision (eg, 5 dB) as a performance outcome [23,29]. Therefore, we added time efficiency as a necessary parameter. Where available, accuracy and reliability were expressed in decibels using the overall root mean square deviation (RMSD) between the automated approach and the gold (or reasonable) standard. On the basis of the study by Margolis et al [30] and the minimum acceptable accuracy recommended by clinical guidelines [31], RMSD values of 6 dB and 10 dB were chosen as criteria for desired and minimal accuracy, respectively. To establish a benchmark for an acceptable test duration, the mean testing time for conventional manual bilateral audiometry (air 7 and bone 5 frequencies) was estimated (Multimedia Appendix 3 [27-29,31,34,38]). For manual bilateral air conduction, based on the benchmark measurement times, a mean testing time of 5 to 10 minutes was considered acceptable, and for manual bilateral air and bone conduction, 10 to 20 minutes was considered acceptable. If testing times exceeded these ranges by >5 minutes, the time efficiency was assessed as a potential issue.

Data collected from the reports provided key information about the scope and details of each report, enabling the authors to assess commonalities between the approaches.

### Collating Approaches, Summarizing, and Reporting the Results

When multiple reports described the same underlying approach, these reports were pooled into one approach cluster. The first report describing an approach and subsequent studies that validated or extended the approach were included. The name of the approach, citations to the initial report, or common authorships were used to cluster the reports. The grading table was completed for each cluster separately to provide a structure for the subsequent content analysis. In the last part of the grading table, under the heading *Validation Approach*, all validation studies are described together. For every approach cluster, a key contribution to the audiological field was derived from the associated reports. A key contribution is a finding or claim made by the authors significant to the approach in general, stated in either the conclusion or the discussion section of a report in accordance with their objective.

## Results

### Overview

A total of 64 reports were included in this study. Of the 64 reports, 56 (88%) were included according to the inclusion and exclusion criteria, and 8 (13%) were added to the approach clusters. After clustering identical approaches, 27 approach clusters remained, including 2 that used machine learning. Extracted data items and grading of results on approaches are provided in Multimedia Appendix 4 [21,23,27-30,32-89]. The specifications of the reported accuracy, reliability, and time efficiency are described in Table 1.

**Table 1:** Review of the accuracy, test–retest reliability, and time efficiency for automated and machine learning audiometry approaches (2012-2021; N=27 approach clusters).

Type of transducer			Accuracy					Reliability (test–retest)					Time efficiency		
			Reported finding		Values, n (%)		Reported finding			Values, n (%)		Reported finding			Values, n (%)
**Air conduction (n=23 approach clusters)**															
			RMSD^a^<6 dB^b^		4 (17)		RMSD<6 dB			4 (17)		Acceptable testing time per (partial) audiogram			10 (43)
			RMSD<10 dB		7 (30)		RMSD<10 dB			1 (4)		Acceptable testing time and number of trials per audiogram			2 (9)
			Statistical equivalence		9 (39)		Statistical equivalence			9 (39)		Acceptable testing time and number of trials per frequency			1 (4)
			No statistical equivalence		3 (13)		Not reported			9 (39)		Testing time potential burden			1 (4)
			N/A^c^		N/A		N/A			N/A		Not reported			9 (39)
**Bone conduction (n=1 approach cluster)**															
			Statistical equivalence		1 (100)		Test–retest not reported			1 (100)		Not reported			1 (100)
**Both air and bone conduction (n=3 approach clusters)**															
	**Air conduction**														
		RMSD<6 dB		2 (67)		RMSD<6 dB			1 (33)		Acceptable testing time per audiogram			2 (67)	
		RMSD<10 dB		1 (33)		RMSD<10 dB			2 (67)		N/A			N/A	
	**Bone conduction**														
		RMSD<10 dB		1 (33)		RMSD<6 dB			1 (33)		N/A			N/A	
		Statistical equivalence		2 (67)		Test–retest not reported			2 (67)		N/A			N/A	
	**Air and bone conduction**														
		N/A		N/A		N/A			N/A		Acceptable testing time per audiogram			1 (33)	

^a^RMSD: root mean square deviation.

^b^dB: decibels.

^c^N/A: not applicable.

### Accuracy

Accuracy is represented as a comparison against the gold standard or reasonable standard. Most of the automated techniques (14/27, 52%) expressed accuracy in RMSD. Other types of analyses used average differences and SD (10/27, 37%), average thresholds and SD (1/27, 4%) [32], linear regression and correlation coefficients (1/27, 4%) [33], and analysis of variance (1/27, 4%) [34]. The types of analysis used can be seen in Multimedia Appendix 5 [23,32-37,39,40,43,45,48-50,57-59, 65,67,68,70,74,77,81,83-85].

### Test–Retest Reliability

Test–retest reliability was reported for some automated and machine learning audiometry approaches. Of the 27 approaches, 17 (63%) did not report on test–retest reliability, and 7 (26%) expressed it in RMSD. Other statistical methods used were average differences and SD (6/27, 22%), Pearson product moment correlation coefficients (2/27, 7%) [35,36], standard of variance (1/27, 4%) [37], and repeated analysis of variance (1/27, 4%) [34].

### Test Efficiency

Of the 27 approaches, 17 (63%) reported a measure for test efficiency based on the test duration. Test efficiency expressed in testing time seems to be a standard metric, similar across studies and defined as the time from presenting the first stimulus until the final response of the participant, expressed in seconds or minutes. However, there were disagreement among reports on what to include in the measurement and what groups to use as a reference. Reported time-efficiency measures included the recorded time per frequency, recorded time per unilateral or bilateral air conduction audiogram (between 2 and 7 frequencies) in normal hearing or people with hearing impairment, or full air and bone conduction audiograms in people with hearing impairment. Of the 27 approach clusters, 13 (48%) approach clusters reported acceptable testing times; 3 (11%) approach clusters indicated the number of trials in addition to the testing time for either a bilaterally masked air audiogram [29], unilateral air audiogram [23], or per frequency [38]; 1 (4%) approach cluster that applied Bekesy tracking reported the testing time but was not in the acceptable range [39]; and 10 (37%) approach clusters did not report anything about the testing time.

### Test Parameters and Specifications

All tests were self-administered from the point at which the test started. Approximately 15% (4/27) of approaches had the option of switching to a manual audiometry mode. Table 2 summarizes an overview of the test parameters and specifications of the 27 approach clusters, and Table 3 highlights the key contributions. Most of the approaches used adaptive procedures that relied only on the previous response (here referred to as partially adaptive procedures).

The most common example was the (modified) Hughson-Westlake staircase procedure (20/27, 74%), which is based on the classical method of limits [91]. Other partially adaptive procedures applied the method of adjustment, such as the Bekesy tracking method [39] or the *coarse-to-fine focus* algorithm [40]. There was a single report of an approach that did not define the threshold-seeking method but had a built-in protocol to alternate between ears during testing [35]. In contrast, fully adaptive procedures used a complete set of all previous responses. Examples include Bayesian active learning procedures (also referred to as machine learning audiometry; 2/27, 7%) [21,23] and maximum likelihood estimation (2/27, 7%) [37,38]. All machine learning audiometry methods applied active Bayesian model selection, which is a type of shallow machine learning that uses individual models. They apply supervised learning, as every data point is labeled by the participant [22].

Most of the approaches (20/27, 74%) used conventional calibration according to the International Organization for Standardization standards. Of the 27 approaches, 6 (22%) used an unconventional calibration technique. Patel et al [32] determined a reference equivalent threshold level for air conduction for a specific phone–headphone combination using manual audiometry as a reference. Masalski et al [41] used reference levels for calibration for smartphone and transducer combinations, collected under uncontrolled conditions in people with normal hearing. Other calibration techniques set the volume of the device to 50% [42], comparing and adjusting the output level to the input using a sound level meter [34,43], or using Thévenin-equivalent probe calibration [39].

Of the 27 approaches, 22 (82%) were validated in people with normal hearing and hearing impairment. Approximately 7% (4/56) of studies were performed in people with normal hearing [34,36,38]. One of the approach clusters was only validated in a population with hearing impairments using hearing aids as transducers [40]. Automated audiometry was applied across a range of populations. All approaches were applied to adults, except in the study by Patel et al [32] that only included children. Approximately 30% (8/27) approaches were validated in children, including 50% (4/8) of approaches that designed a child-friendly user interface [32,44-46]. Other test populations were older people [47], veterans [48], and persons exposed to occupational noise [49] or ototoxic substances [50]. Automated audiometry has also been applied as an alternative to traditional manual audiometry in low-resource environments [51-53]. The user interface plays an important role in making self-testing feasible in all populations and may require an iterative design process (including clinical pilot studies) [52,54].

**Table 2:** Description of test parameters and specifications for automated audiometry approaches (2012-2021; N=27).

Test parameters and specifications			Descriptions of approach clusters, n (%)
**Threshold-seeking method (underlying algorithm to determine the thresholds)**			
	Hughson-Westlake (modified)	20 (74)	
	Machine learning	2 (7)	
	Bekesy tracking	1 (4)	
	Other method	4 (15)	
**Test range (limits of the frequency that can be tested)**			
	Clinical frequency range (125 Hz-8000 Hz)	18 (67)	
	Extended high frequencies range (125 Hz-16,000 Hz)	4 (15)	
	Reduced frequency range	5 (19)	
**Test range (limits of intensity that can be tested)**			
	Intensity range (0-100 dB^a^ hearing level)	14 (52)	
	Reduced intensity range	10 (37)	
	Intensity range not reported	3 (11)	
**Masking (needed to prevent responses from the nontest ear and obtain the true threshold of the test ear)**			
	Automated masking	9 (33)	
	Manual masking	1 (4)	
	No masking	13 (48)	
	Masking not reported	4 (15)	
**Response method (method of recording participants’ responses to test stimuli)**			
	Forced choice	9 (33)	
	Single response	13 (48)	
	Forced choice and single response	3 (11)	
	Not reported	2 (7)	
**Transducers (method of presenting stimuli, eg, insert phone or supra- or circumaural headphones)**			
	Air conduction transducers	23 (85)	
	Air and bone conduction transducers	3 (11)	
	Only bone conduction transducer	1 (4)	
**Calibration (unconventional calibration methods are explained in the text)**			
	Conventional calibration	20 (74)	
	Unconventional calibration	6 (22)	
	Calibration not reported	1 (4)	
**Digital devices (reported hardware needed to run the test)**			
	Portable audiometer	2 (7)	
	Computer based	9 (33)	
	Web-based (requires connectivity)	1 (4)	
	Smartphone- or tablet-based	1 (4)	
**Quality control measures (indicators of the reliability of the test)**			
	Detect false responses	5 (19)	
	Have noise control	6 (22)	
	Detect false responses and have noise control	7 (26)	
	Quality control measures not reported	9 (33)	
**Validation (highest level of validation reported for each approach cluster)**			
	Gold standard	22 (82)	
	Reasonable standard	4 (15)	
	Proof of concept	1 (4)	
**Test population (hearing status)**			
	Normal hearing only	3 (11)	
	Hearing loss only	1 (4)	
	Normal hearing and hearing loss	23 (85)	
**Test population (age)**			
	Adults only	17 (63)	
	Children only	1 (4)	
	Adults and children	9 (33)	

^a^dB: decibels

**Table 3:** Key contributions of the automated and machine learning approaches to the audiological field.

Approach cluster (lead author of first report, reports)	Approach cluster (name)	Key contributions to the field
Bean et al [55]	OtoKiosk	It has the potential to be used in test environments such as examination rooms as a clinical tool for identifying hearing loss via air conduction separating people with normal and impaired hearing.
Chen et al [40]	SHSA^a^	It is a hearing test that runs on a hearing aid, which has statistical equivalence to manual audiometry.
Colsman et al [36]	—^b^	Portable devices that use calibrated headphones result in much higher accuracies than uncalibrated devices.
Corry et al [34]	—	The reliability of audiometer apps should not be assumed. Issues of accuracy and calibration of consumer headphones need to be addressed before such combinations can be used with confidence.
Dewyer et al [33]	Earbone	It is a proof of concept for smartphone-based bone conduction threshold testing.
Foulad et al [43,51,56]	Eartrumpet	It is an iOS-based software app for automated pure-tone hearing testing without the need for additional specialized equipment, yielding hearing test results that approach those of conventional audiometry.
Jacobs et al [50,57]	Oto-ID	They are automated (remote) hearing tests to provide clinicians information for ototoxicity monitoring.
Kung et al [45]	Kids Hearing Game	It includes tablet-based audiometry using game design elements that can be used to test and screen for hearing loss in children who may not have adequate access to resources for a traditional hearing screening.
Liu et al [58]	—	A self-testing system comprising a notebook computer, sound card, and insert earphones is a valid, portable, and sensitive instrument for hearing thresholds self-assessment.
Manganella et al [35]	Agilis	It is an application that detects increased levels of ambient noise when it is programmed to stop the testing.
Margolis et al [30,46,59-61]	AMTAS^c^	AMTAS is designed to fit into the clinical care pathway, including air and bone conduction, and incorporates a quality assessment method (QUALIND) that predicts the accuracy of the test.
Margolis et al [48,62,63]	Home Hearing Test	It is developed and well-suited to provide increased access to hearing testing and support home telehealth programs.
Masalski and Krecicki [41,64,65]	—	It is an automated method that uses smartphone model–specific reference sound levels for calibration in the app. Biological reference sound levels were collected in uncontrolled conditions in people with normal hearing.
Meinke et al [66,67]	WHATS^d^	WHATS is a mobile wireless automated hearing test system in occupational audiometry for obtaining hearing thresholds in diverse test locations without the use of a sound booth.
Patel et al [32]	HearTest^e^	It is a novel, subjective, test-based approach used to calibrate a smartphone–earphone combination with respect to the reference audiometer.
Poling et al [39]	—	Specific Bekesy tracking patterns were identified in people who experienced difficulty converging to a reliable threshold.
Schlittenlacher et al [23]	—	Bayesian active learning methods provide an accurate estimate of hearing thresholds in a continuous range of frequencies.
Schmidt et al [37]	—	A user-operated, 2-alternative, forced choice in combination with the method of maximum likelihood does not require specific operating skills; repeatability is acceptable and is similar to conventional audiometry.
Song et al [21,29,68,69]	MLAG^f^	MLAG is a Bayesian active learning method that determines the most informative next tone, leading to a fast audiogram procedure and threshold estimation in a continuous range of frequencies, with the potential to measure additional variables efficiently.
Sun et al [70]	—	It is an active noise control technology to measure outside the sound booth.
Swanepoel et al [27,47,53,71-75]	KUDUwave	It is an automated portable diagnostic audiometer using improved passive attenuation and real-time environmental noise monitoring, making audiometry possible in unconventional settings.
Swanepoel et al [28,52,54,76-80]	HearTest^g^	It is a smartphone-based automated hearing test applicable in low-resource environments.
Szudek et al [42,81,82]	Uhear	It is an approach that is applicable to the initial evaluation of patients with sudden sensorineural hearing loss before a standard audiogram is available.
Van Tasell and Folkeard [83]	—	Method of adjustment and the Hughson–Westlake method embedded in automated audiometry can be considered equivalent in accuracy to conventional audiometry.
Vinay et al [38,49]	NEWT^h^	NEWT, which is incorporated inside an active communication earplug, serves as a reliable and efficient method of measuring auditory thresholds, especially in the presence of high background noise.
Whitton et al [84]	—	It is a proof-of-concept study of several self-administered, automated hearing measurements at home, showing statistical equivalency to conventional audiometry in the clinic.
Yeung et al [44,85-89]	Shoebox	It is a method for threshold hearing assessments outside conventional sound booths and with an interface suitable for children.

^a^SHSA: smartphone-based hearing self-assessment.

^b^Not available.

^c^AMTAS: Automated Method for Testing Auditory Sensitivity.

^d^WHATS: Wireless Automated Hearing Test System.

^e^Smartphone-based hearing test app (not yet commercialized).

^f^MLAG: Machine Learning Audiogram.

^g^Automated hearing test commercialized by the hearX group.

^h^NEWT: The New Early Warning Test.

## Discussion

### Principal Findings

In 2013, evidence for automated audiometry demonstrated similar reliability and accuracy as that of manual audiometry. However, especially for children and bone conduction, the number of reports was limited [24]. In less than a decade, 22 novel approaches and developments across 5 existing approaches had appeared in 56 publications, adding to the 29 papers published before 2013. Promising new developments include the use of machine learning techniques for more time-efficient hearing assessment (2/27, 7%), use of tablets or smartphones as audiometer interface (15/27, 56%), and child-friendly user interfaces (4/27, 15%), including game design elements. The number of approaches that include bone conduction is still limited (4/27, 15%)—only 7% (2/29) more approaches were reported compared with the number reported in 2013 [24].

### Accuracy

The required accuracy, reliability, and efficiency depend on the clinical aims and consequences. The ultimate aim of the automated hearing assessment is to deliver clinically actionable estimates of hearing status (ie, the clinician or patient acts appropriately for treatment, given the diagnostic test results). In fully adaptive procedures, the level of precision and confidence needed to conclude the assessment can be set to any level by choosing the proper termination criteria, resulting in different trade-offs. A study by Schmidt et al [37], for instance, aimed for high accuracy and reliability, whereas a study by Heisey et al [29] aimed for high efficiency with machine learning audiometry. Overall, a shift in the type of analysis to demonstrate the accuracy has been observed. In this review, the 2 major types of analysis included were RMSD (14/27, 52%) and average differences and SD (10/27, 37%). In the report by Mahomed et al [24], accuracy was primarily expressed in average differences (11/27, 41%) or thresholds and SD (11/27, 41%). In our view, RMSD is the preferred indicator for accuracy as it has clinical relevance [31], assuming it has already been demonstrated that there is no bias between the automated and manually determined hearing thresholds (eg, signed differences). In traditional clinical terms, automation is equal in accuracy to manual audiometry if the difference is within 6 dB RMSD. Of the 27 automated approaches, 6 (22%) meet this strict accuracy criterion. However, for many applications, the less strict 10 dB RMSD criterium is sufficient, which was achieved by 26% (7/27) additional automated approaches.

For bone conduction measurements, the accuracy was inherently lower than that of air conduction measurements because of conductor placement [30]. However, this reduced accuracy is typically sufficient to address the clinical question of whether conductive or mixed hearing loss is present, as well as choose and evaluate appropriate treatment. The technical feasibility of bone conduction assessments outside of a clinical setting (sound booth) remains difficult. Alternatively, this clinical question can be addressed with other tests, including tympanometry, otoscopy, or a combination of air conduction thresholds for tone and speech stimuli [90]. At least 13 automated techniques had accuracy comparable with that of traditional manual air conduction audiometry, as expressed in RMSD.

A limitation to the impact of achieved test accuracy is the high variation in the interpretation of audiograms by clinicians, regardless of whether those audiograms are determined using an automated or manual approach [92]. Automation can assist clinicians and patients in interpreting the measurement by data-driven automated reporting of accuracy and reliability (including signaling for suspicious outcomes) such as QUALIND [60] or by automated classification for diagnostic purposes (including the type and degree of hearing loss). Examples of automated classification include AMCLASS [93], Autoaudio [94], and data-driven audiogram classification [95].

### Reliability

RMSD is also increasingly used as a measure of test–retest reliability. Of the 27 approaches that reported test–retest reliability, 8 (30%) used RMSD as a measure, whereas in 2013, this was only used in 2 (2/29, 7%) studies. Furthermore, 41% (11/27) of approaches did not report on test–retest reliability or used a measure of statistical equivalence that did not allow us to assess the accuracy. Advances in automated audiometry that increase reliability include procedures to identify invalid responses (5/27, 19%), monitoring environmental noise (6/27, 22%), or both (7/27, 26%) to warn for invalid test conditions, making these tests applicable in more populations and environments. The reliability can be increased, for instance, by alternative response methods, including the forced-choice paradigm [37], or by using machine learning to account for lapses of attention [23]. Digital (health) technologies, including smartphones and tablets, lend themselves to quality control measures for increased reliability with the host of integrated sensors [6].

### Efficiency

A fair indicator of efficiency is the overall time required to conduct a test. Most approaches (20/27, 74%) used the modified Hughson–Westlake procedure, of which some (7/20, 35%) showed a similar test duration to manual audiometry. Maximum likelihood procedures demonstrated a 45% reduction in test time in people with normal hearing [38]. Bayesian active learning methods can be extended by adding variables that share some interrelationships using a conjoint estimator that exploits nonlinear interactions between the variables [96]. The resulting machine learning–based automated procedures demonstrated a 30% to 70% reduction in test time compared with manual audiometry for air conduction audiograms in people with normal hearing and hearing impairment [29]. No machine learning approaches had incorporated bone conduction. Therefore, time-efficiency gains compared with full audiogram procedures are not available; however, one can assume that these will yield similar time-efficiency gains. Another indicator of test efficiency is the number of stimuli required to achieve the desired accuracy. This indicator is helpful in optimizing the threshold-seeking part of the approach. Reporting the equivalent time gains under operational conditions is recommended as this can be readily compared with other efficiency gains, including the reduced traveling time if a visit to the outpatient clinic can be replaced for an at-home test or time savings by automating other parts of the clinical care pathway such as interpretation of the outcome. Other aspects of efficiency beyond time that should be considered are cost reductions when enabling task shifting of professionals or the ability to test outside the sound booth.

### Future Developments

To obtain an overall indicator of the technical maturity of an approach, developers should be encouraged to use the technology readiness level (TRL) to report the development phase of a technology. TRLs were initially developed in the aerospace industry to estimate the maturity of technology from basic concepts to flight-proven products [97]. To apply TRLs to automated audiometry, further adjustments can be made to fit the hearing health care sector to the version of biomedical TRLs created by the US Army Medical Research and Materiel Command [98]. For those approaches that are ready for operational use, certification (eg, Conformité Européenne and the United States Food and Drug Administration) can further stimulate clinical adoption and iterative improvements based on clinical feedback. In order to be cost-effective, timely, and responsive, certification for digital self-care approaches may need to be less stringent than those for clinical care. A study by Yeung et al [12] proposed alternative procedures for (fast) certification to keep up with the rapidly developing field of visual eHealth tools. Their recommendations might also be applicable to automated hearing assessments, including a rating by health agencies or nongovernmental organizations (eg, a repository of trusted approaches; see Psyberguide [99] as an example of mental health apps reviewed by experts) or adopting the Clinical Laboratory Improvement Amendments model to ensure that approaches comply with the basic requirements of usability, privacy, and security [12]. Following similar certification procedures in the visual and auditory domains may facilitate diagnosis across medical domains. In addition, standards on minimum quality and consensus on what metadata are needed in health applications to describe the test conditions and facilitate interpretation are currently missing.

### Limitations

This scoping review included peer-reviewed reports from widely used and recognized scientific databases. A potential limitation is that some of the commercialized automated approaches may have been developed without peer-reviewed reports. Therefore, some automated approaches could be more mature than previously reported. There is no gold standard for reporting audiometry validation studies, which limits a consistent comparison among approaches. Finally, automated procedures may well be embraced by early adopters first, which could lead to projections on suitability that are overly optimistic for users with poorer digital proficiency.

### Conclusions and Recommendations

Since 2013, an increasing number of automated audiometry approaches on digital devices have demonstrated similar accuracy, reliability, and time efficiency as conventional manual audiometry. New developments offer features, versatility, and cost-effectiveness beyond manual audiometry. Fully adaptive procedures, including machine learning techniques, seek hearing thresholds more efficiently. Inexpensive digital devices such as smartphones can be turned into audiometers, increasing accessibility and availability. Higher reliability is achievable by signaling invalid test conditions, and child-friendly user interfaces offer a solution to the hard-to-test population. These approaches can be implemented in the clinical care pathway, remote or virtual hearing health care, community-based services, and occupational health care to address the global need for accessible hearing loss diagnosis.

For successful adoption, standardized measures of accuracy, reliability, and efficiency are needed for comparative purposes. Certification and independent reviews may help prospective users select trustworthy approaches. Further reliability can be achieved by determining which difficult-to-test populations may not be appropriate for automated testing and how to detect and then triage these patients to specialized centers. More user-friendly and failsafe procedures that include remote surveillance and quality control can support automated hearing assessment at scale in specific populations and in concert with diagnostic assessments in other medical domains, including visual health and mental well-being [12,99]. Further contextual information, such as standardized metadata, is needed to help clinicians interpret the context and limitations of test outcomes. If researchers and clinicians deal carefully with their limitations, automated hearing assessments can be designed such that they form an effective part of service delivery for many people who have or are at risk of hearing loss. Automated audiometry can be part of existing care pathways and also enable new service models, including task shifting to community health workers delivering decentralized care, virtual hearing health care, and over-the-counter or direct-to-consumer hearing aid dispensing.

## Appendix

Multimedia Appendix 1 Search strategy.

Multimedia Appendix 2 Template table for grading approaches.

Multimedia Appendix 3 Estimated mean testing time for conventional manual bilateral audiometry.

Multimedia Appendix 4 Graded approaches.

Multimedia Appendix 5 Types of statistical analyses for accuracy and reliability.

## Abbreviations

PRISMA: Preferred Reporting Items for Systematic Reviews and Meta-Analyses
RMSD: root mean square deviation
TRL: technology readiness level
