# Supplementary material for: Digital Approaches to Automated and Machine Learning Assessments of Hearing: Scoping Review
Source: J Med Internet Res. 2022 Feb 2;24(2):e32581. doi: 10.2196/32581 (PMC8851345; doi:10.2196/32581)
Supplement: Multimedia Appendix 1 [file jmir_v24i2e32581_app1.docx]

**Supplementary Table 1: Search strategy**

| **PubMed** | “Machine learning” [Mesh] OR “mobile applications” [Mesh] OR “neural network” [tiab] OR “algorithm” [tiab] OR “DNN-based” [tiab] OR “data-driven” [tiab] OR “supervised Learning” [tiab] OR “learning-based” [tiab] OR “Automatic” [tiab] OR “Autonomous” [tiab] OR “computerized” [tiab] OR “computerized” [tiab] OR “computer-based” [tiab] OR "pc-based" [tiab] OR "application-based” [tiab] OR “device-based” [tiab] OR "automation" [tiab] OR "automated" [tiab] OR “audioscan*” [tiab] OR “Machine learning” [tiab] OR “MLAG” [tiab] OR “smartphone*” [tiab] OR "tablet" [tiab] OR "iPhone" [tiab] OR "iPad" [tiab] OR "iOS" [tiab] OR "android" [tiab] OR “mobile device” OR portable [tiab] OR "self-test" [tiab] OR "online" [tiab] OR “active learning” [tiab] OR ”transfer learning“ [tiab] OR ”gaussian process*“ [tiab] OR ”Bayesian classifi*“ [tiab] OR "home-based" [tiab] OR "remote*" [tiab] OR "remote care" [tiab] OR "home*" [tiab] OR "telehealth" [tiab] OR "teleaudiology" [tiab] OR "e-health" [tiab] OR "ehealth" [tiab] OR "m-health" [tiab] OR "mhealth" [tiab] |
| --- | --- |
|  | AND |
|  | “auditory threshold” [Mesh] OR “Audiometry” [Mesh] OR "audiometry" [tiab] OR "hearing measurement*" [tiab] OR "hearing threshold*" [tiab] OR "auditory threshold*" [tiab] OR "hearing assessment*" [tiab] OR "hearing evaluation"[tiab] OR “hearing test*” [tiab] OR “hearing perception level” [tiab] OR “Audition assessment” [tiab] OR “audiometric*” [tiab] OR “audiometer“ [tiab] OR “audiogram” [tiab] |
| **IEEE** | "auditory threshold" OR "Audiometry" OR "audiometry" OR "hearing measurement*" OR "hearing threshold*" OR "auditory threshold*" OR "hearing assessment*" OR "hearing evaluation" OR "hearing test*" OR "hearing perception level" OR "Audition assessment" OR "audiometric*" OR "audiometer "OR "audiogram" OR "hearing loss" OR "hearing ability" |
| **Web of Science** | "Machine learning"  OR "mobile applications"  OR "neural network"  OR "algorithm"  OR "DNN-based"  OR "data-driven"  OR "supervised Learning"  OR "learning-based" OR "Automatic"  OR "Autonomous"  OR "computerized"  OR "computerized"  OR "computer-based"  OR "pc-based"  OR "application-based"  OR "device-based"  OR "automation"  OR "automated"  OR "audioscan*"  OR "Machine learning"  OR "MLAG"  OR "smartphone*"  OR "tablet"  OR "iPhone"  OR "iPad"  OR "iOS"  OR "android"  OR "mobile device"  OR portable  OR "self-test"  OR "online"  OR "active learning"  OR "transfer learning"  OR "gaussian process*"  OR "Bayesian classifi* "  OR "home-based"  OR "remote*"  OR "remote care"  OR "home*"  OR "telehealth"  OR "teleaudiology"  OR "e-health"  OR "ehealth"  OR "m-health"  OR "mhealth" |
|  | AND |
|  | "auditory threshold"  OR "audiometry"  OR "hearing measurement*"  OR "hearing threshold*"  OR "auditory threshold*"  OR "hearing assessment*"  OR "hearing evaluation"  OR "hearing test*"  OR "hearing perception level"  OR "Audition assessment"  OR "audiometric*"  OR "audiometer"  OR "audiogram" |

PubMed, IEEE and Web of Science were searched for relevant studies. All available MeSH terms were combined with free text words of all known synonyms of *automated/machine learning* and *audiometry*.
