# Supplementary material for: Digital Approaches to Automated and Machine Learning Assessments of Hearing: Scoping Review
Source: J Med Internet Res. 2022 Feb 2;24(2):e32581. doi: 10.2196/32581 (PMC8851345; doi:10.2196/32581)
Supplement: Multimedia Appendix 2 [file jmir_v24i2e32581_app2.docx]

**Supplementary table 2: template table for grading reports**

| **Introduction** [short description of the approach]  **Selected reports:** [reports included in the search]  **Additional resources:** [including reports added based on the reference list of selected reports and validation studies from before the search time window] | |
| --- | --- |
| **Threshold Seeking Method and Range** | |
| **Frequencies** | Clinical [octave frequencies 125-8000 Hz] / high-resolution [additional in-between frequencies] /reduced / extended range [>8000 Hz] /not reported |
| **Intensities** | clinical [-10 – 120 dBHL] / reduced [10-100 dBHL]/ not reported |
| **Masking** | automated / manual / other / no / not reported |
| **Seeking approach** | (modified) Hughson-Westlake / Machine learning [Bayesian Active Learning procedure] / Bekesy tracking [continuous tracking of audible sound]/ other [including maximum likelihood estimation] |
| **Response Method and Representation of Results** | |
| **Response method** | forced choice [e.g. forced choice two alternative approach] / single response [e.g. stair case method] / not reported |
| **Test paradigm** | self-test [self-administered from the point the test starts] / facilitated by operator [The operator can be a layman when the task is limited to initializing a test and supervision or can be an expert if one reverts to manual testing] |
| **Representation of results** | conventional [i.e. clinical audiogram] / high-resolution representation [fine-feature audiogram] / automated classification [provides an indication of the severity and type of hearing loss] / not reported |
| **Test Equipment** | |
| **Transducers** | air conduction/ bone conduction  (**type**), [could by any type of transducer including a hearing aid] |
| **Calibration** | conventional calibration according to ISO-standard xx] / unconventional [e.g based on a reference group of normal hearing subject measured with the same equipment, or Thévenin-equivalent probe calibration [26] / no / not reported |
| **Hardware** | portable audiometer / computer-based / web-based / smartphone / tablet |
| **Test Quality Control** | |
| **Comparison automated versus manual** | Root-Mean-Square-Deviation (RMSD) versus manual approach for all frequencies < 10 dB or < 6 dB / statistical equivalence [e.g. paired *t*-test, intraclass correlation coefficients] / statistically not equivalent / not reported |
| **Test-retest** | RMSD < 10 dB / < 6 dB [retest accuracy for automated audiometry between sessions]/ statistical equivalence / statistically not equivalent / not reported |
| **Deals with** | false-responses [i.e. false positives, false negatives, attention lapses or cheating]/ noise control [i.e. background noise monitoring, noise cancellation or noise suppression]/ both |
| **Validation Approach, Test Population, and Context** | |
| **Validation** | gold standard / reasonable standard  xx subjects included, age range subject (reference) |
| **Test Population** | Normal hearing [hearing thresholds better than 20 dB HL] / hearing loss / children [age below 16 years] / adults / veterans / low-resource environment / ototoxic- / self- / noise- monitoring / infectious disease |
| **Efficiency** | Testing time / number of stimuli / not reported  mean testing time xx minutes, range xx minutes, per frequency / for automated / manual partial audiogram / unilateral / bilateral air and bone conduction audiogram in normal hearing / hearing impaired. RMSD fell below xx dB after xx trials. |

Template table for grading reports. The text in between brackets [] explains the criteria for used in the grading process.
