# Supplementary material for: Digital Approaches to Automated and Machine Learning Assessments of Hearing: Scoping Review
Source: J Med Internet Res. 2022 Feb 2;24(2):e32581. doi: 10.2196/32581 (PMC8851345; doi:10.2196/32581)
Supplement: Multimedia Appendix 3 [file jmir_v24i2e32581_app3.docx]

**Supplementary Material Table 3: Estimated Mean Testing Time for Conventional Manual Bilateral Audiometry**

| **Reference** | [27] | [38] | [34] | [28] | [28] | [29] | [29] |
| --- | --- | --- | --- | --- | --- | --- | --- |
| **Number of subjects** | 30 | 22 | 20 | 30 | 61 | 8 | 10 |
| **Transducers** | Air | Air | Air | Air | Air | Air &  bone | Air &  bone |
| **Number of frequencies** | 7 | 4 | 6 | 5 | 5 | 6 (air)  5 (bone) | 6 (air)  5 (bone) |
| **Masking** | No | No | No | No | No | Yes | Yes |
| **Hearing status of subjects** | NH | NH | NH | HL | NH | HL | HL  (asymmetry) |
| **Age (years)** | 18-31 | 25-37 | 21-26 | 24-92 | 16-21 | 21-83 | 21-83 |
| **Mean testing times** |  |  |  |  |  |  |  |
| **air conduction (minutes)** | 7.7 | 4.3 | 4.5 | 6.7 | 3.2 | 6.9 | 9.9 |
| **bone conduction (minutes)** | - | - | - | - | - | 3.8 | 8.6 |
| **Air and bone (minutes)** |  |  |  |  |  | 10.7 | 18.5 |
| **Standard deviation** | 2.3 | 2 | - | 2.5 | 0.6 | - | - |
| **Min** | 4.2 | - | - | - | - | - | - |
| **Max** | 12.6 | - | - | - | - | - | - |
| **time per frequency** | 1.1 | 1.1 | 0.8 | 1.3 | 0.6 | 1.2 | 1.7 |

Table 4: Testing times for manual audiometry in normal hearing (NH) and Hearing Impaired (HL) listeners.

The mean testing time for conventional manual audiometry was estimated based on studies that reported testing time [27-29,34,38]. Testing time was defined as the time from presenting the first stimulus to the first ear until the last subject response for the second ear, excluding the time needed for test instruction and placing the transducer [27] . Studies reported the mean testing time for a 4-7 frequency manual unilateral or bilateral audiogram. Therefore, testing times have been extrapolated to a standard audiogram (seven air and 5 bone frequencies measured bilaterally) based on the ASHA guidelines [31]. Table 4 provides the reported testing times and primary characteristics per study. In case of a unilateral audiogram the time was doubled to estimate a bilateral audiogram [38]. All studies applied the modified Hughson-Westlake procedure.

The estimated mean testing time for a standard air audiogram is 5.8 minutes based on 133 normal hearing subjects [27,28,34,38]. In hearing impaired persons, the estimated mean testing time is 9.6 minutes based on 48 subjects [28,29]. Test duration increased in cases of asymmetry [29]. For air and bone conduction in hearing impaired only Heisey et al. [29] reported test times, resulting in a mean testing time of 14.5 minutes based on 18 subjects. Bone conduction took 3.8 to 8.6 minutes in hearing impaired subjects. No studies were found that recorded testing time in normal hearing.
