# Supplementary material for: Digital Approaches to Automated and Machine Learning Assessments of Hearing: Scoping Review
Source: J Med Internet Res. 2022 Feb 2;24(2):e32581. doi: 10.2196/32581 (PMC8851345; doi:10.2196/32581)
Supplement: Multimedia Appendix 4 [file jmir_v24i2e32581_app4.docx]

**Supplementary Table 4: Graded Approaches**

1. Bean et al, OtoKiosk

| **Introduction** Otokiosk is an iOS-based system developed for clinical hearing assessment  **Selected reports:** Bean et al., 2021 [55]  **Additional reports:** none | |
| --- | --- |
| **Threshold Seeking Method and Range** | |
| **Frequencies** | clinical / high-resolution /***reduced*** / extended range /not reported |
| **Intensities** | clinical / reduced / ***not reported*** |
| **Masking** | automated /manual / other / no / ***not reported*** |
| **Seeking approach** | ***(modified) Hughson-Westlake*** / MLAG / Bekesy tracking / other |
| **Response Method and Presentation of Results** | |
| **Response method** | forced choice ***/ single response*** / not reported |
| **Test paradigm** | ***self-test*** / facilitated by operator |
| **Presentation of results** | ***conventional /*** high-resolution representation / automated classification / not reported |
| **Test Equipment** | |
| **Transducers** | ***air conduction***/ bone conduction  (Peltor H7A earmuffs with RadioEar DD45 transducer) |
| **Calibration** | ***conventional*** / unconventional / no / not reported |
| **Hardware** | portable audiometer / computer-based / web-based / smartphone/***tablet*** |
| **Test Quality Control** | |
| **Comparison automated versus manual** | ***RMSD < 10 dB*** / < 6 dB / statistical equivalence / statistically not equivalent / not reported |
| **Test-retest** | RMSD < 10 dB / < 6 dB / statistical equivalence / statistically not equivalent / ***not reported*** |
| **Deals with** | false-responses / ***noise control*** |
| **Validation Approach, Test Population, and Context** | |
| **Validation** | ***gold standard*** / reasonable standard / proof of concept  26 subjects included, 47-76 years |
| **Test Population** | ***Normal hearing / hearing loss***/ children / ***adults*** / elderly / veterans / low-resource environment / ototoxic- / self- / noise- monitoring / infectious disease |
| **Efficiency** | Testing time / number of stimuli / ***not reported*** |

1. Chen et al

| **Introduction:** smartphone-based hearing self-assessment system using hearing aids with fast audiometry method (SHSA)  **Selected reports:** Chen et al., 2019 [40]  **Additional reports:** none | |
| --- | --- |
| **Threshold Seeking Method and Range** | |
| **Frequencies** | ***clinical*** / high-resolution /reduced / extended range /not reported |
| **Intensities** | ***clinical*** / extended / reduced / not reported |
| **Masking** | automated /manual / other / ***no*** / not reported |
| **Seeking approach** | (modified) Hughson-Westlake / MLAG / Bekesy tracking / ***other*** |
| **Response Method and Presentation of Results** | |
| **Response method** | forced choice / ***single response*** / not reported |
| **Test paradigm** | ***self-test*** / facilitated by operator |
| **Presentation of results** | ***conventional*** / high-resolution representation / automated classification / not reported |
| **Test Equipment** | |
| **Transducers** | ***air conduction***/ bone conduction  (hearing aids) |
| **Calibration** | ***conventional*** / unconventional / no / not reported |
| **Hardware** | portable audiometer / computer-based / web-based / ***smartphone*** / tablet |
| **Test Quality Control** | |
| **Comparison automated versus manual** | ***RMSD*** < 10 dB / ***< 6 dB*** / statistical equivalence / statistically not equivalent / not reported |
| **Test-retest** | RMSD < 10 dB / < 6 dB / statistical equivalence / statistically not equivalent / ***not reported*** |
| **Deals with** | false-responses / ***noise control*** |
| **Validation Approach, Test Population, and Context** | |
| **Validation** | ***gold standard*** / reasonable standard / proof of concept  20 subjects included, 34-73 years |
| **Test Population** | Normal hearing / ***hearing loss*** / children / ***adults*** / elderly / veterans / low-resource environment / ototoxic- / self- / noise- monitoring / infectious disease |
| **Efficiency** | ***Testing time*** / number of stimuli / not reported  mean testing time 0.7-1.3 minutes for automated unilateral air conduction audiogram in normal hearing and hearing impaired  mean testing time 1.5-3 minutes for manual unilateral air conduction audiogram in normal hearing and hearing impaired |

1. Colsman et al

| **Introduction:** a calibrated app for pure-tone screening audiometry by self-assessment on a tablet  **Selected reports:** Colsman et al., 2020 [36]  **Additional reports:** none | |
| --- | --- |
| **Threshold Seeking Method and Range** | |
| **Frequencies** | ***clinical*** / high-resolution /reduced / extended range /not reported |
| **Intensities** | ***clinical*** / extended / reduced / not reported |
| **Masking** | automated /manual / other / ***no*** / not reported |
| **Seeking approach** | ***(modified) Hughson-Westlake*** / MLAG / Bekesy tracking / other |
| **Response Method and Presentation of Results** | |
| **Response method** | ***forced choice*** / single response / not reported |
| **Test paradigm** | ***self-test*** / facilitated by operator |
| **Presentation of results** | ***conventional*** / high-resolution representation / automated classification / not reported |
| **Test Equipment** | |
| **Transducers** | ***air conduction***/ bone conduction  (Sennheiser HDA 280) |
| **Calibration** | ***conventional*** / unconventional / no / not reported |
| **Hardware** | portable audiometer / computer-based / web-based / smartphone/ ***tablet*** |
| **Test Quality Control** | |
| **Comparison automated versus manual** | ***RMSD < 10 dB*** / < 6 dB / statistical equivalence / statistically not equivalent / not reported |
| **Test-retest** | RMSD < 10 dB / < 6 dB / ***statistical equivalence*** / statistically not equivalent / not reported |
| **Deals with** | false-responses / noise control |
| **Validation Approach, Test Population, and Context** | |
| **Validation** | ***gold standard*** / reasonable standard / proof of concept  68 subjects included, age range 19-65 years |
| **Test Population** | ***Normal hearing*** / hearing loss / children / ***adults*** / elderly / veterans / low-resource environment / ototoxic- / self- / noise- monitoring / infectious disease |
| **Efficiency** | ***Testing time*** / number of stimuli / not reported  median testing time 12 minutes, range 10-20 minutes, for automated bilateral air conduction audiogram in normal hearing. |

1. Corry et al

| **Introduction:** app-based audiometer using commercial earphones  **Selected reports:** Corry et al., 2017 [34]  **Additional reports:** none | |
| --- | --- |
| **Threshold Seeking Method and Range** | |
| **Frequencies** | ***clinical*** / high-resolution /reduced / extended range /not reported |
| **Intensities** | ***clinical*** / extended / reduced / not reported |
| **Masking** | automated /***manual*** / other / no / not reported |
| **Seeking approach** | ***(modified) Hughson-Westlake*** / MLAG / Bekesy tracking / other |
| **Response Method and Presentation of Results** | |
| **Response method** | forced choice / ***single response*** / not reported |
| **Test paradigm** | ***self-test*** / ***facilitated by operator*** |
| **Presentation of results** | ***conventional*** / high-resolution representation / automated classification / not reported |
| **Test Equipment** | |
| **Transducers** | ***air conduction***/ bone conduction  (commercial earbuds) |
| **Calibration** | conventional / ***unconventional*** / no / not reported |
| **Hardware** | portable audiometer / computer-based / web-based / smartphone/***tablet*** |
| **Test Quality Control** | |
| **Comparison automated versus manual** | RMSD < 10 dB / < 6 dB / statistical equivalence / ***statistically not equivalent*** / not reported |
| **Test-retest** | RMSD < 10 dB / < 6 dB / ***statistical equivalence*** / statistically not equivalent / not reported |
| **Deals with** | ***false-responses*** / noise control |
| **Validation Approach, Test Population, and Context** | |
| **Validation** | ***gold standard*** / reasonable standard / proof of concept  20 subjects included, 21-26 years |
| **Test Population** | ***Normal hearing*** / hearing loss / children / ***adults*** / elderly / veterans / low-resource environment / ototoxic- / self- / noise- monitoring / infectious disease |
| **Efficiency** | **Testing time** / number of stimuli / not reported  Mean testing time 4 minutes for bilateral air conduction audiogram in normal hearing (4.5 minutes manually). |

1. Dewyer et al, Earbone

| **Introduction:** an automated smartphone app that determines bone-conduction pure-tone thresholds  **Selected reports:** Dewyer et al., 2019 [33]  **Additional reports:** none | |
| --- | --- |
| **Threshold Seeking Method and Range** | |
| **Frequencies** | clinical / high-resolution /***reduced*** / extended range /not reported |
| **Intensities** | clinical / extended / ***reduced*** / not reported |
| **Masking** | automated /manual / other / ***no*** / not reported |
| **Seeking approach** | ***(modified) Hughson-Westlake*** / MLAG / Bekesy tracking / other |
| **Response Method and Presentation of Results** | |
| **Response method** | forced choice ***/ single response*** / not reported |
| **Test paradigm** | ***self-test*** / facilitated by operator |
| **Presentation of results** | ***conventional*** / high-resolution representation / automated classification / not reported |
| **Test Equipment** | |
| **Transducers** | air conduction/ ***bone conduction***  (Radioear B71 bone oscillator) |
| **Calibration** | ***conventional*** / unconventional / no / not reported |
| **Hardware** | portable audiometer / computer-based / web-based / ***smartphone***/tablet |
| **Test Quality Control** | |
| **Comparison automated versus manual** | RMSD < 10 dB / < 6 dB / ***statistical equivalence*** / statistically not equivalent / not reported |
| **Test-retest** | RMSD < 10 dB / < 6 dB / statistical equivalence / statistically not equivalent / ***not reported*** |
| **Deals with** | false-responses / noise control |
| **Validation Approach, Test Population, and Context** | |
| **Validation** | gold standard / reasonable standard / ***proof of concept***  80 subjects included, 24-82 years |
| **Test Population** | ***Normal hearing / hearing loss*** / children / ***adults*** / elderly / veterans / low-resource environment / ototoxic- / self- / noise- monitoring / infectious disease |
| **Efficiency** | Testing time / number of stimuli / ***not reported*** |

1. Foulad et al, Eartrumpet

| **Introduction:** Eartrumpet is an iOS-based automated hearing testing application  **Selected reports:** Foulad et al., 2013 [43,51,56]  **Additional reports:** none | |
| --- | --- |
| **Threshold Seeking Method and Range** | |
| **Frequencies** | ***clinical*** / high-resolution /reduced / extended range /not reported |
| **Intensities** | clinical / extended / ***reduced*** / not reported |
| **Masking** | ***automated*** /manual / other / no / not reported |
| **Seeking approach** | ***(modified) Hughson-Westlake*** / MLAG / Bekesy tracking / other |
| **Response Method and Presentation of Results** | |
| **Response method** | forced choice / ***single response*** / not reported |
| **Test paradigm** | ***self-test*** / facilitated by operator |
| **Presentation of results** | ***conventional*** / high-resolution representation / automated classification / not reported |
| **Test Equipment** | |
| **Transducers** | ***air conduction***/ bone conduction  (Bose QuietComfort 15 Acoustic noise cancelling headphones and consumer earbuds) |
| **Calibration** | conventional / ***unconventional*** / no / not reported |
| **Hardware** | portable audiometer / computer-based / web-based / ***smartphone/tablet*** |
| **Test Quality Control** | |
| **Comparison automated versus manual** | ***RMSD < 10 dB*** / < 6 dB / statistical equivalence / statistically not equivalent / not reported |
| **Test-retest** | RMSD < 10 dB / < 6 dB / statistical equivalence / statistically not equivalent / ***not reported*** |
| **Deals with** | false-responses / noise control |
| **Validation Approach, Test Population, and Context** | |
| **Validation** | ***gold standard*** / reasonable standard / proof of concept  42 subjects included, 20-85 years [43],  35 subjects included, 19-85 years [51],  33 subjects included, 18-65 years [56] |
| **Test Population** | ***Normal hearing / hearing loss*** / children / ***adults*** / elderly / veterans / low-resource environment / ototoxic- / self- / noise- monitoring / infectious disease |
| **Efficiency** | ***Testing time*** / number of stimuli / not reported  mean testing time 5 minutes for automated bilateral air conduction audiogram in normal hearing and hearing impaired [56]. |

1. Jacobs et al, Oto-ID

| **Introduction:** A portable audiometer for ototoxicity monitoring  **Selected reports:** Jacobs et al., 2012 [50,57]  **Additional reports:** none | |
| --- | --- |
| **Threshold Seeking Method and Range** | |
| **Frequencies** | ***clinical /*** high-resolution /reduced */* ***extended range*** /not reported |
| **Intensities** | ***clinical*** / extended / reduced / not reported |
| **Masking** | automated /manual / other / ***no*** / not reported |
| **Seeking approach** | ***(modified) Hughson-Westlake*** / MLAG / Bekesy tracking / other |
| **Response Method and Presentation of Results** | |
| **Response method** | forced choice / ***single response*** / not reported |
| **Test paradigm** | ***self-test*** / facilitated by operator |
| **Presentation of results** | ***conventional / high-resolution representation*** / automated classification / not reported |
| **Test Equipment** | |
| **Transducers** | ***air conduction***/ bone conduction  (HDA200) |
| **Calibration** | ***conventional*** / unconventional / no / not reported |
| **Hardware** | ***portable audiometer*** / computer-based / web-based / smartphone/tablet |
| **Test Quality Control** | |
| **Comparison automated versus manual** | ***RMSD*** ***< 10 dB*** / < 6 dB / statistical equivalence / statistically not equivalent / not reported |
| **Test-retest** | ***RMSD < 10 dB /*** < 6 dB / statistical equivalence / statistically not equivalent / not reported |
| **Deals with** | ***false-responses / noise control*** |
| **Validation Approach, Test Population, and Context** | |
| **Validation** | ***gold standard*** / reasonable standard / proof of concept  9 subjects included, 8-24 years [50],  40 subjects included, 8-74 years [57] |
| **Test Population** | ***Normal hearing / hearing loss*** / children / ***adults*** / elderly / veterans / low-resource environment / ototoxic- / self- / noise- monitoring / infectious disease |
| **Efficiency** | Testing time / number of stimuli / ***not reported*** |

1. Kung et al

| **Introduction** Kids Hearing Game (KHG) determines an audiogram using a game format.  **Selected reports:** Kung et al., 2021 [45]  **Additional reports:** none | |
| --- | --- |
| **Threshold Seeking Method and Range** | |
| **Frequencies** | ***clinical*** / high-resolution /reduced / extended range /not reported |
| **Intensities** | clinical / ***reduced*** / not reported |
| **Masking** | automated /manual / other / no / ***not reported*** |
| **Seeking approach** | ***(modified) Hughson-Westlake*** / MLAG / Bekesy tracking / other |
| **Response Method and Presentation of Results** | |
| **Response method** | forced choice / ***single response*** / not reported |
| **Test paradigm** | ***self-test*** / facilitated by operator |
| **Presentation of results** | conventional / high-resolution representation / automated classification / ***not reported*** |
| **Test Equipment** | |
| **Transducers** | ***air conduction***/ bone conduction  (Ausdom F01 wired over-ear headphone) |
| **Calibration** | ***conventional*** / unconventional / no / not reported |
| **Hardware** | portable audiometer / computer-based / web-based / smartphone/***tablet*** |
| **Test Quality Control** | |
| **Comparison automated versus manual** | RMSD < 10 dB / < 6 dB / ***statistical equivalence*** / statistically not equivalent / not reported |
| **Test-retest** | RMSD < 10 dB / < 6 dB / statistical equivalence / statistically not equivalent / ***not reported*** |
| **Deals with** | ***false-responses*** / noise control |
| **Validation Approach, Test Population, and Context** | |
| **Validation** | ***gold standard*** / reasonable standard / proof of concept  18 subjects included, 6-11 years |
| **Test Population** | ***Normal hearing/ hearing loss/ children*** / adults / elderly / veterans / low-resource environment / ototoxic- / self- / noise- monitoring / infectious disease |
| **Efficiency** | Testing time / number of stimuli / ***not reported*** |

1. Liu et al

| **Introduction:** software-based hearing self-testing system  **Selected reports:** Liu et al., 2015 [58]  **Additional reports:** none | |
| --- | --- |
| **Threshold Seeking Method and Range** | |
| **Frequencies** | ***clinical*** / high-resolution /reduced / extended range /not reported |
| **Intensities** | ***clinical*** / extended / reduced / not reported |
| **Masking** | ***automated*** /manual / other / no / not reported |
| **Seeking approach** | ***(modified) Hughson-Westlake*** / MLAG / Bekesy tracking / other |
| **Response Method and Presentation of Results** | |
| **Response method** | ***forced choice*** / single response / not reported |
| **Test paradigm** | ***self-test*** / facilitated by operator |
| **Presentation of results** | ***conventional*** / high-resolution representation / automated classification / not reported |
| **Test Equipment** | |
| **Transducers** | ***air conduction***/ bone conduction  (ER-3A) |
| **Calibration** | ***conventional*** / unconventional / no / not reported |
| **Hardware** | portable audiometer / ***computer-based*** / web-based / smartphone/tablet |
| **Test Quality Control** | |
| **Comparison automated versus manual** | RMSD < 10 dB / < 6 dB / ***statistical equivalence*** / statistically not equivalent / not reported |
| **Test-retest** | RMSD < 10 dB / < 6 dB / statistical equivalence / statistically not equivalent / ***not reported*** |
| **Deals with** | false-responses / noise control |
| **Validation Approach, Test Population, and Context** | |
| **Validation** | gold standard / ***reasonable standard*** / proof of concept  50 subjects included, age range not reported |
| **Test Population** | ***Normal hearing / hearing loss*** / children / ***adults*** / elderly / veterans / low-resource environment / ototoxic- / self- / noise- monitoring / infectious disease |
| **Efficiency** | Testing time / number of stimuli / ***not reported*** |

1. Manganella et al

| **Introduction:** a tablet-based Agilis Health Mobile Audiogram as an effective and valid measure of hearing thresholds in an adult and paediatric population. <http://www.agilishealth.com/>  **Selected reports:** Manganella et al., 2018 [35]  **Additional reports:** none | |
| --- | --- |
| **Threshold Seeking Method and Range** | |
| **Frequencies** | ***clinical*** / high-resolution /reduced / extended range /not reported |
| **Intensities** | clinical / extended / reduced / ***not reported*** |
| **Masking** | automated /manual / other / ***no*** / not reported |
| **Seeking approach** | (modified) Hughson-Westlake / MLAG / Bekesy tracking / ***other*** |
| **Response Method and Presentation of Results** | |
| **Response method** | ***forced choice*** / single response / not reported |
| **Test paradigm** | ***self-test*** / facilitated by operator |
| **Presentation of results** | ***conventional*** / high-resolution representation / automated classification / not reported |
| **Test Equipment** | |
| **Transducers** | ***air conduction***/ bone conduction  (Etymotic HF5 earphones) |
| **Calibration** | ***conventional*** / unconventional / no / not reported |
| **Hardware** | portable audiometer / computer-based / web-based / smartphone/***tablet*** |
| **Test Quality Control** | |
| **Comparison automated versus manual** | RMSD < 10 dB / < 6 dB / ***statistical equivalence*** / statistically not equivalent / not reported |
| **Test-retest** | RMSD < 10 dB / < 6 dB / ***statistical equivalence*** / statistically not equivalent / not reported |
| **Deals with** | ***false-responses / noise control*** |
| **Validation Approach, Test Population, and Context** | |
| **Validation** | ***gold standard*** / reasonable standard / proof of concept  27 subjects included, 12+ years |
| **Test Population** | ***Normal hearing / hearing loss / children / adults*** / elderly / veterans / low-resource environment / ototoxic- / self- / noise- monitoring / infectious disease |
| **Efficiency** | ***Testing time*** / number of stimuli / not reported  mean testing time 10 minutes, range 8–14 minutes, for automated bilateral air conduction audiogram in normal hearing and hearing impaired |

1. Margolis et al, AMTAS

| **Introduction:** AMTAS is commercialized by audiology incorporated and sold by Grason Stadlor (GSI). More info can be found on: http://audiologyincorporated.com/about  **Selected reports:** Eikelboom et al., 2013 [59]  **Additional reports:** Margolis et al., 2007 [30,46,60,61] | |
| --- | --- |
| **Threshold Seeking Method and Range** | |
| **Frequencies** | ***clinical*** / high-resolution /reduced / extended range /not reported |
| **Intensities** | clinical / extended / ***reduced*** / not reported |
| **Masking** | ***automated*** /manual / other / no / not reported |
| **Seeking approach** | ***(modified) Hughson-Westlake*** / MLAG / Bekesy tracking / other |
| **Response Method and Presentation of Results** | |
| **Response method** | ***forced choice*** / single response / not reported |
| **Test paradigm** | ***self-test / facilitated by operator*** |
| **Presentation of results** | conventional / high-resolution representation / ***automated classification*** / not reported |
| **Test Equipment** | |
| **Transducers** | ***air conduction/ bone conduction***  (Earphone TDH200 and Radioear B71) |
| **Calibration** | ***conventional*** / unconventional / no / not reported |
| **Hardware** | portable audiometer / ***computer-based*** / web-based / smartphone/tablet |
| **Test Quality Control** | |
| **Comparison automated versus manual** | ***RMSD*** < 10 dB / ***< 6 dB*** / statistical equivalence / statistically not equivalent / not reported |
| **Test-retest** | ***RMSD*** < 10 dB / ***< 6 dB*** / statistical equivalence / statistically not equivalent / not reported |
| **Deals with** | ***false-responses / noise control*** |
| **Validation Approach, Test Population, and Context** | |
| **Validation** | ***gold standard***  30 subjects included, age range not reported [30],  96 subjects, 4-8 years and adults [46],  120 subjects included, 21-65 years [60],  13 subjects included, 21-65 years [61]  ***reasonable standard*** / proof of concept  44 subjects included, 21-88 years [59] |
| **Test Population** | ***Normal hearing / hearing loss/*** ***children / adults*** / elderly / veterans / low-resource environment / ototoxic- / self- / noise- monitoring / infectious disease |
| **Efficiency** | ***Testing time*** / number of stimuli / not reported  mean testing time 7 minutes, range 6–8 minutes, for automated bilateral air and bone conduction audiogram in normal hearing [30]  mean testing time 11 minutes, range 6–21 minutes, for automated bilateral air and bone conduction audiogram in hearing impaired [29]  mean testing time 16 minutes, range 10–31 minutes, for automated bilateral air and bone conduction audiogram in hearing impaired [59] |

1. Margolis et al, HHT

| **Introduction:** Home hearing test is an AMTAS based test procedure that subjects can carry out at home. The home hearing test supports only air conduction. Distributed by Etymotic Research  **Selected reports:** Margolis et al., 2016 [48,62,63]  **Additional reports:** none | |
| --- | --- |
| **Threshold Seeking Method and Range** | |
| **Frequencies** | clinical / high-resolution /***reduced*** / extended range /not reported |
| **Intensities** | clinical / extended / ***reduced*** / not reported |
| **Masking** | automated /manual / other / ***no*** / not reported |
| **Seeking approach** | ***(modified) Hughson-Westlake*** / MLAG / Bekesy tracking / other |
| **Response Method and Presentation of Results** | |
| **Response method** | ***forced choice*** / single response / not reported |
| **Test paradigm** | ***self-test*** / facilitated by operator |
| **Presentation of results** | conventional / high-resolution representation /***automated classification*** / not reported |
| **Test Equipment** | |
| **Transducers** | ***air conduction***/ bone conduction  (Earphones modified Etymotic Research mc5) |
| **Calibration** | ***conventional*** / unconventional / no / not reported |
| **Hardware** | portable audiometer / ***computer-based*** / web-based / smartphone/tablet |
| **Test Quality Control** | |
| **Comparison automated versus manual** | ***RMSD*** < 10 dB / ***< 6 dB*** / statistical equivalence / statistically not equivalent / not reported |
| **Test-retest** | ***RMSD*** < 10 dB / ***< 6 dB*** / statistical equivalence / statistically not equivalent / not reported |
| **Deals with** | ***false-responses / noise control*** |
| **Validation Approach, Test Population, and Context** | |
| **Validation** | ***gold standard***  126 subjects included, 44-88 years [62],  112 subject included, 60+ years [63]  ***reasonable standard*** ***/*** proof of concept  28 subjects included, 44-88 years [48] |
| **Test Population** | ***Normal hearing / hearing loss/*** children / ***adults*** / elderly / ***veterans*** / low-resource environment / ototoxic- / self- / noise- monitoring / infectious disease |
| **Efficiency** | ***Testing time*** / number of stimuli / not reported  mean testing time 10 minutes, for automated bilateral air conduction audiogram in hearing impaired [63] |

1. Masalski et al

| **Introduction:** A smartphone-based automated hearing test biologically calibrated. Calibration of mobile devices conducted by means of a biological method involves determining reference sound level in relation to the hearing threshold of normal-hearing persons  **Selected reports:** Masalski et al, 2013 [41,64,65]  **Additional reports:** none | |
| --- | --- |
| **Threshold Seeking Method and Range** | |
| **Frequencies** | ***clinical*** / high-resolution /reduced / extended range /not reported |
| **Intensities** | ***clinical*** / extended / reduced / not reported |
| **Masking** | ***automated*** /manual / other / no / not reported |
| **Seeking approach** | ***(modified) Hughson-Westlake*** / MLAG / Bekesy tracking / other |
| **Response Method and Presentation of Results** | |
| **Response method** | ***forced choice*** / single response / not reported |
| **Test paradigm** | ***self-test*** / facilitated by operator |
| **Presentation of results** | ***conventional*** / high-resolution representation / automated classification / not reported |
| **Test Equipment** | |
| **Transducers** | ***air conduction***/ bone conduction  (type not reported) |
| **Calibration** | conventional / ***unconventional*** / no / not reported |
| **Hardware** | portable audiometer / computer-based / web-based / ***smartphone*** / tablet |
| **Test Quality Control** | |
| **Comparison automated versus manual** | RMSD < 10 dB / < 6 dB / statistical equivalence / ***statistically not equivalent*** / not reported |
| **Test-retest** | RMSD < 10 dB / < 6 dB / ***statistical equivalence*** / statistically not equivalent / not reported |
| **Deals with** | false-responses / noise control |
| **Validation Approach, Test Population, and Context** | |
| **Validation** | ***gold standard*** / reasonable standard / proof of concept  51 subjects included, 11-60 years [64],  70 subjects included, 18-71 years [65],  number of subjects and age range not reported [41] |
| **Test Population** | ***Normal hearing / hearing loss*** / children / ***adults*** / elderly / veterans / low-resource environment / ototoxic- / self- / noise- monitoring / infectious disease |
| **Efficiency** | Testing time / number of stimuli / ***not reported*** |

1. Meinke et al, WHATS

| **Introduction:** Creare is a wireless audiometer developed by Creare LLC. Wireless automated hearing test system. **Selected reports:** Meinke et al., 2017 [66,67]  **Additional reports:** none | |
| --- | --- |
| **Threshold Seeking Method and Range** | |
| **Frequencies** | ***clinical*** / high-resolution /reduced / extended range /not reported |
| **Intensities** | ***clinical*** / extended / reduced / not reported |
| **Masking** | automated /manual / other / ***no*** / not reported |
| **Seeking approach** | ***(modified) Hughson-Westlake*** / MLAG / Bekesy tracking / other |
| **Response Method and Presentation of Results** | |
| **Response method** | forced choice / ***single response*** / not reported |
| **Test paradigm** | ***self-test*** / facilitated by operator |
| **Presentation of results** | ***conventional*** / high-resolution representation / automated classification / not reported |
| **Test Equipment** | |
| **Transducers** | ***air conduction***/ bone conduction  (headphone) |
| **Calibration** | ***conventional*** / unconventional / no / not reported |
| **Hardware** | portable audiometer / ***computer-based*** / web-based / smartphone/tablet |
| **Test Quality Control** | |
| **Comparison automated versus manual** | RMSD < 10 dB / < 6 dB / ***statistical equivalence*** / statistically not equivalent / not reported |
| **Test-retest** | RMSD < 10 dB / < 6 dB / ***statistical equivalence*** / statistically not equivalent / not reported |
| **Deals with** | false-responses / noise control |
| **Validation Approach, Test Population, and Context** | |
| **Validation** | gold standard ***/ reasonable standard*** / proof of concept  20 subjects included, 18+ years [67];  101 subjects included, 6-9 years [66] |
| **Test Population** | ***Normal hearing / hearing loss / children / adults*** / elderly / veterans / low-resource environment / ototoxic- / self- / noise- monitoring / infectious disease |
| **Efficiency** | ***Testing time*** / number of stimuli / not reported  mean testing time 20 minutes, for subsequent automated and manual bilateral air audiogram in normal hearing and hearing impaired children [66] |

1. Patel et al

| **Introduction** The HearTest application runs on an iPhone (8/10/XR) combined with Sennheiser CX300 earphones  **Selected reports:** Patel et al., 2021 [32]  **Additional reports:** none | |
| --- | --- |
| **Threshold Seeking Method and Range** | |
| **Frequencies** | ***clinical*** / high-resolution /reduced / extended range /not reported |
| **Intensities** | clinical / ***reduced*** / not reported |
| **Masking** | automated /manual / other / ***no*** / not reported |
| **Seeking approach** | ***(modified) Hughson-Westlake*** / MLAG / Bekesy tracking / other |
| **Response Method and Presentation of Results** | |
| **Response method** | ***forced choice*** / single response / not reported |
| **Test paradigm** | ***self-test*** / facilitated by operator |
| **Presentation of results** | ***conventional*** / high-resolution representation ***/ automated classification*** / not reported |
| **Test Equipment** | |
| **Transducers** | ***air conduction***/ bone conduction  (Sennheiser CX300 earphone) |
| **Calibration** | conventional / ***unconventional*** / no / not reported |
| **Hardware** | portable audiometer / computer-based / web-based / ***smartphone***/tablet |
| **Test Quality Control** | |
| **Comparison automated versus manual** | RMSD < 10 dB / < 6 dB / ***statistical equivalence*** / statistically not equivalent / not reported |
| **Test-retest** | RMSD < 10 dB / < 6 dB / statistical equivalence / statistically not equivalent / ***not reported*** |
| **Deals with** | false-responses / ***noise control*** |
| **Validation Approach, Test Population, and Context** | |
| **Validation** | ***gold standard*** / reasonable standard / proof of concept  14 subjects included, 20-30 years |
| **Test Population** | ***Normal hearing*** / hearing loss/ children / ***adults*** / elderly / veterans / low-resource environment / ototoxic- / self- / noise- monitoring / infectious disease |
| **Efficiency** | Testing time / number of stimuli / ***not reported*** |

1. Poling et al

| **Introduction:** a comparison of a clinical modified Hughson-Westlake (manual) method with an automated, modified (single frequency) Békésy tracking method  **Selected reports:** Poling et al., 2016 [39]  **Additional reports:** none | |
| --- | --- |
| **Threshold Seeking Method and Range** | |
| **Frequencies** | clinical / high-resolution /reduced / ***extended range*** /not reported |
| **Intensities** | ***clinical*** / extended / reduced / not reported |
| **Masking** | automated /manual / other / ***no*** / not reported |
| **Seeking approach** | (modified) Hughson-Westlake / MLAG / ***Bekesy tracking*** / other |
| **Response Method and Presentation of Results** | |
| **Response method** | forced choice / ***single response*** / not reported |
| **Test paradigm** | ***self-test*** / facilitated by operator |
| **Presentation of results** | ***conventional*** / high-resolution representation / automated classification / not reported |
| **Test Equipment** | |
| **Transducers** | ***air conduction*** / bone conduction  (not reported) |
| **Calibration** | conventional / ***unconventional*** / no / not reported |
| **Hardware** | ***portable audiometer*** / computer-based / web-based / smartphone/tablet |
| **Test Quality Control** | |
| **Comparison automated versus manual** | RMSD < 10 dB / < 6 dB / ***statistical equivalence*** / statistically not equivalent / not reported |
| **Test-retest** | RMSD < 10 dB / < 6 dB / ***statistical equivalence*** / statistically not equivalent / not reported |
| **Deals with** | false-responses / noise control |
| **Validation Approach, Test Population, and Context** | |
| **Validation** | ***gold standard*** / reasonable standard / proof of concept  10 subjects included, 19- 47 years |
| **Test Population** | ***Normal hearing / hearing loss*** / children / ***adults*** / elderly / veterans / low-resource environment / ototoxic- / self- / noise- monitoring / infectious disease |
| **Efficiency** | ***Testing time*** / number of stimuli / not reported  mean testing time 17 minutes for automated unilateral air conduction audiogram in normal hearing and hearing impaired (11-13 minutes manually) |

1. Schlittenlacher et al

| **Introduction:**  Two methods, a counting method and yes/no task, for estimating audiograms quickly and accurately using Bayesian active learning  **Selected reports:** Schlittenlacher et al., 2018 [23]  **Additional reports:** none | |
| --- | --- |
| **Threshold Seeking Method and Range** | |
| **Frequencies** | ***clinical / high-resolution*** /reduced / extended range /not reported |
| **Intensities** | clinical / extended / ***reduced*** / not reported |
| **Masking** | automated /manual / other / ***no*** / not reported |
| **Seeking approach** | (modified) Hughson-Westlake / ***MLAG*** / Bekesy tracking / other |
| **Response Method and Presentation of Results** | |
| **Response method** | ***forced choice / single response*** / not reported |
| **Test paradigm** | ***self-test*** / facilitated by operator |
| **Presentation of results** | ***conventional*** / high-resolution representation / automated classification / not reported |
| **Test Equipment** | |
| **Transducers** | ***air conduction***/ bone conduction  (HDA200) |
| **Calibration** | ***conventional*** / unconventional / no / not reported |
| **Hardware** | portable audiometer / ***computer-based*** / web-based / smartphone/tablet |
| **Test Quality Control** | |
| **Comparison automated versus manual** | ***RMSD < 10 dB*** / < 6 dB / statistical equivalence / statistically not equivalent / not reported |
| **Test-retest** | ***RMSD <*** 10 dB / ***< 6 dB*** / statistical equivalence / statistically not equivalent / not reported |
| **Deals with** | ***false-responses*** / noise control |
| **Validation Approach, Test Population, and Context** | |
| **Validation** | ***gold standard*** / reasonable standard / proof of concept  20 subjects included, 21-77 years |
| **Test Population** | ***Normal hearing / hearing loss***/ children / ***adults*** / elderly / veterans / low-resource environment / ototoxic- / self- / noise- monitoring / infectious disease |
| **Efficiency** | ***Testing time*** / ***number of stimuli*** / not reported  Mean testing time 4 minutes for automated unilateral air audiogram in normal hearing and hearing impaired. RMSD fell below 5 dB after 18 trials for the Counting method and this criterion was after 28 trials for the Yes/No method. |

1. Schmidt et al

| **Introduction:** a user-operated audiometry method based on the maximum likelihood principle  **Selected reports:** Schmidt et al., 2014 [37]  **Additional reports:** none | |
| --- | --- |
| **Threshold Seeking Method and Range** | |
| **Frequencies** | ***clinical*** / high-resolution /reduced / extended range /not reported |
| **Intensities** | ***clinical*** / extended / reduced / not reported |
| **Masking** | automated /manual / other / ***no*** / not reported |
| **Seeking approach** | (modified) Hughson-Westlake / MLAG / Bekesy tracking / ***other*** |
| **Response Method and Presentation of Results** | |
| **Response method** | ***forced choice*** / single response / not reported |
| **Test paradigm** | ***self-test*** / facilitated by operator |
| **Presentation of results** | ***conventional*** / high-resolution representation / automated classification / not reported |
| **Test Equipment** | |
| **Transducers** | ***air conduction***/ bone conduction  (HAD 200) |
| **Calibration** | ***conventional*** / unconventional / no / not reported |
| **Hardware** | portable audiometer / ***computer-based*** / web-based / smartphone/tablet |
| **Test Quality Control** | |
| **Comparison automated versus manual** | RMSD < 10 dB / < 6 dB / ***statistical equivalence*** / statistically not equivalent / not reported |
| **Test-retest** | RMSD < 10 dB / < 6 dB / ***statistical equivalence*** / statistically not equivalent / not reported |
| **Deals with** | ***false-responses*** / noise control |
| **Validation Approach, Test Population, and Context** | |
| **Validation** | ***gold standard*** / reasonable standard / proof of concept  41 subjects included, 11-69 years |
| **Test Population** | ***Normal hearing / hearing loss /*** ***children / adults*** / elderly / veterans / low-resource environment / ototoxic- / self- / noise- monitoring / infectious disease |
| **Efficiency** | ***Testing time*** / ***number of stimuli*** / not reported  Mean testing time 2 minutes per frequency for automated air conduction audiogram in normal hearing and hearing impaired. Participants needed  on average 36 trials to complete a frequency. |

1. Song et al

| **Introduction** Machine Learning audiometry (MLAG) is currently available as a research tool used on a small scale in clinics. MLAG is commercialized by [Bonauria](http://www.bonauria.com/).  **Selected reports:** Song et al., 2015 [21,29,68,69]  **Additional reports:** none | |
| --- | --- |
| **Threshold Seeking Method and Range** | |
| **Frequencies** | ***clinical / high-resolution*** /reduced / extended range /not reported |
| **Intensities** | ***clinical*** / extended / reduced /not reported |
| **Masking** | ***automated*** /manual / other / no / not reported |
| **Seeking approach** | (modified) Hughson-Westlake / ***MLAG*** / Bekesy tracking / other |
| **Response Method and Presentation of Results** | |
| **Response method** | forced choice / ***single response*** / not reported |
| **Test paradigm** | ***self-test*** / facilitated by operator |
| **Presentation of results** | ***conventional / high-resolution representation*** / automated classification / not reported |
| **Test Equipment** | |
| **Transducers** | ***air conduction***/ bone conduction  (HDA200, TDH-39) |
| **Calibration** | ***conventional*** / unconventional / no / not reported |
| **Hardware** | portable audiometer / computer-based / ***web-based*** / smartphone/tablet |
| **Test Quality Control** | |
| **Comparison automated versus manual** | ***RMSD*** < 10 dB / ***< 6 dB*** / statistical equivalence / statistically not equivalent / not reported |
| **Test-retest** | ***RMSD*** < 10 dB / ***< 6 dB*** / statistical equivalence / statistically not equivalent / not reported |
| **Deals with** | ***false-responses*** / noise control |
| **Validation Approach, Test Population, and Context** | |
| **Validation** | ***gold standard***  21 subjects included, 18-90 years [68],  21 subject included, 19-79 years [21],  29 subjects included, 29-83 years [29]  ***Reasonable standard*** /proof of concept  34 subjects included, age range not reported [69] |
| **Test Population** | ***Normal hearing*** / ***hearing loss***/ children / **adults** / elderly / veterans / low-resource environment / ototoxic- / self- / noise- monitoring / infectious disease |
| **Efficiency** | ***Testing time*** / ***number of stimuli*** / not reported  RMSD fell below 5 dB after a median of 67 trials using an automated Hughson-Westlake algorithm for unilateral air audiogram in normal hearing and hearing impaired. The MLAG algorithm 49 trials for the same accuracy [21].  Mean testing time 2.1-4.8 minutes for bilateral air audiogram in normal hearing. RMSD fell below 5 dB after 19-44 trials.  Mean testing time 5-7 minutes, for bilateral air audiogram in hearing impaired. RMSD fell below 5 dB after 50-63 trials [29]. |

1. Sun et al

| **Introduction** An automated hearing test equipment based on active noise control technology  **Selected reports:** Sun et al., 2019 [70]  **Additional reports:** none | |
| --- | --- |
| **Threshold Seeking Method and Range** | |
| **Frequencies** | clinical / high-resolution /***reduced*** / extended range /not reported |
| **Intensities** | clinical / extended / reduced /***not reported*** |
| **Masking** | automated /manual / other / no / ***not reported*** |
| **Seeking approach** | ***(modified) Hughson-Westlake*** / MLAG / Bekesy tracking / other |
| **Response Method and Presentation of Results** | |
| **Response method** | forced choice / single response / ***not reported*** |
| **Test paradigm** | ***self-test*** / facilitated by operator |
| **Presentation of results** | conventional / high-resolution representation / ***automated classification*** / not reported |
| **Test Equipment** | |
| **Transducers** | ***air conduction***/ bone conduction  (type not reported) |
| **Calibration** | conventional / unconventional / no / ***not reported*** |
| **Hardware** | portable audiometer / ***computer-based*** / web-based / smartphone/tablet |
| **Test Quality Control** | |
| **Comparison automated versus manual** | ***RMSD < 10 dB*** / < 6 dB / statistical equivalence / statistically not equivalent / not reported |
| **Test-retest** | RMSD < 10 dB / < 6 dB / statistical equivalence / statistically not equivalent / ***not reported*** |
| **Deals with** | ***false-responses / noise control*** |
| **Validation Approach, Test Population, and Context** | |
| **Validation** | ***gold standard*** / reasonable standard / proof of concept  95 subjects included, 20-68 years |
| **Test Population** | ***Normal hearing / hearing loss*** / children / ***adults*** / elderly / veterans / low-resource environment / ototoxic- / self- / noise- monitoring / infectious disease |
| **Efficiency** | Testing time / number of stimuli / ***not reported*** |

1. Swanepoel et al, KUDUwave

| **Introduction** Kuduwave is commercialized by emoyo. For more info see https://emoyo.net/kuduwave/  **Selected reports:** Storey et al., 2014 [53,71,73–75]  **Additional reports:** Swanepoel et al., 2010 [27,47,72] | |
| --- | --- |
| **Threshold Seeking Method and Range** | |
| **Frequencies** | ***clinical / high-resolution*** /reduced / extended range /not reported |
| **Intensities** | ***clinical*** / extended / reduced /not reported |
| **Masking** | ***automated*** /manual / other / no / not reported |
| **Seeking approach** | ***(modified) Hughson-Westlake*** / MLAG / Bekesy tracking / other |
| **Response Method and Presentation of Results** | |
| **Response method** | forced choice / ***single response*** / not reported |
| **Test paradigm** | ***self-test / facilitated by operator*** |
| **Presentation of results** | conventional / high-resolution representation / ***automated classification*** / not reported |
| **Test Equipment** | |
| **Transducers** | ***air conduction/ bone conduction***  (insert earphone, B-71 bone oscillator) |
| **Calibration** | ***conventional*** / unconventional / no / not reported |
| **Hardware** | portable audiometer / ***computer-based*** / web-based / smartphone/tablet |
| **Test Quality Control** | |
| **Comparison automated versus manual** | ***RMSD*** < 10 dB / ***< 6 dB*** / statistical equivalence / statistically not equivalent / not reported |
| **Test-retest** | ***RMSD < 10 dB*** / < 6 dB / statistical equivalence / statistically not equivalent / not reported |
| **Deals with** | ***false-responses /*** ***noise control*** |
| **Validation Approach, Test Population, and Context** | |
| **Validation** | ***gold standard***  38 subjects included, 18-31 years [27],  30 subjects included, 19-77 years [72],  20 subjects included, 19-63 years [53];  50 subjects included, 6-13 years [73],  31 subjects included, 15-80 years [74]  ***reasonable standard*** / proof of concept  147 subjects included, 65-94 years [47],  23 subjects included, 20-75 years [75],  42 subjects included, 19-92 years [71] |
| **Test Population** | ***Normal hearing/ hearing loss/ children /*** ***adults*** / ***elderly*** / veterans / ***low-resource environment*** / ototoxic- / self- / noise- monitoring / infectious disease |
| **Efficiency** | ***Testing time*** / number of stimuli / not reported  mean testing time 7-8 minutes, range 4-13 minutes, both for automated and manual bilateral air conduction audiogram in normal hearing [26] |

1. Swanepoel et al, Heartest

| **Introduction:** Heartest has CE certification. Started as a hearing screening tool (validated hearScreen application), subsequently further developed for diagnostic purposes. [77]  **Selected reports:** Swanepoel et al., 2014 [28,52,54,76–80]  **Additional reports:** none | |
| --- | --- |
| **Threshold Seeking Method and Range** | |
| **Frequencies** | ***clinical / high-resolution*** /reduced / extended range /not reported |
| **Intensities** | clinical / extended / ***reduced*** / not reported |
| **Masking** | ***automated*** /manual / other / no / not reported |
| **Seeking approach** | ***(modified) Hughson-Westlake*** / MLAG / Bekesy tracking / other |
| **Response Method and Presentation of Results** | |
| **Response method** | ***forced choice / single response*** / not reported |
| **Test paradigm** | ***self-test / facilitated by operator*** |
| **Presentation of results** | conventional / high-resolution representation/ ***automated classification*** / not reported |
| **Test Equipment** | |
| **Transducers** | ***air conduction***/ bone conduction  (circumaural headphones Sennheiser HD 202 II /300and insert earphones) |
| **Calibration** | ***conventional*** / unconventional / no / not reported |
| **Hardware** | portable audiometer / computer-based / web-based / ***smartphone/tablet*** |
| **Test Quality Control** | |
| **Comparison automated versus manual** | ***RMSD < 10 dB*** / < 6 dB / statistical equivalence / statistically not equivalent / not reported |
| **Test-retest** | ***RMSD*** < 10 dB / ***< 6 dB*** / statistical equivalence / statistically not equivalent / not reported |
| **Deals with** | ***false-responses / noise control*** |
| **Validation Approach, Test Population, and Context** | |
| **Validation** | ***gold standard***  162 subjects included, 5-8 years [77],  64 subjects included, 18-88 years [54],  95 subjects included, 24-92 years [28],  61 subjects included, 16-64 years [76],  340 subjects included, 5-92 years [79],  232 subjects included, age range not reported [80];  ***reasonable standard*** / proof of concept  30 subjects included, 18-88 years [54],  200 subjects included, 18-55 years [78],  63 subjects included, 20-88 years [52] |
| **Test Population** | ***Normal hearing / hearing loss / children / adults*** / elderly / veterans / low-resource environment /ototoxic- / self- / ***noise- monitoring*** / i***nfectious disease*** |
| **Efficiency** | ***Testing time*** / number of stimuli / not reported  Mean testing time 7 minutes for automated and manual bilateral air conduction audiogram in normal hearing and hearing impaired [28].  Mean testing time 4.5 minutes, range 2 – 14 minutes for automated bilateral partial (PTA) air conduction audiogram in normal hearing and hearing impaired [78]  Mean testing time 5 minutes for automated bilateral air conduction audiogram in normal hearing and hearing impaired in a sound booth, and 6 minutes outside a sound booth [54] |

1. Szudek et al, Uhear

| **Introduction:** Developed by Unitron (<https://www.unitron.com/content/unitron/za/en/professional/practice-support/uhear.html>)  **Selected reports:** Handzel et al., 2013 [81,82]  **Additional reports:** Szudek et al., 2012 [42] | |
| --- | --- |
| **Threshold Seeking Method and Range** | |
| **Frequencies** | ***clinical*** / high-resolution /reduced / extended range /not reported |
| **Intensities** | ***clinical*** / extended / reduced / not reported |
| **Masking** | automated /manual / other / no / ***not reported*** |
| **Seeking approach** | ***(modified) Hughson-Westlake*** / MLAG / Bekesy tracking / other |
| **Response Method and Presentation of Results** | |
| **Response method** | forced choice / single response / ***not reported*** |
| **Test paradigm** | ***self-test*** / facilitated by operator |
| **Presentation of results** | conventional / high-resolution representation / automated classification / ***not reported*** |
| **Test Equipment** | |
| **Transducers** | ***air conduction***/ bone conduction  (Creative EP 630 earbuds) |
| **Calibration** | conventional /***unconventional*** / no / not reported |
| **Hardware** | portable audiometer / computer-based / web-based / ***smartphone/tablet*** |
| **Test Quality Control** | |
| **Comparison automated versus manual** | RMSD < 10 dB / < 6 dB / statistical equivalence / ***statistically not equivalent*** / not reported |
| **Test-retest** | RMSD < 10 dB / < 6 dB / statistical equivalence / statistically not equivalent / ***not reported*** |
| **Deals with** | false-responses / noise control |
| **Validation Approach, Test Population, and Context** | |
| **Validation** | ***gold standard*** / reasonable standard / proof of concept  32 subjects included, 20-82 years [81],  68 subjects included, 8-10 years [82],  100 subjects included, 18+ years [42] |
| **Test Population** | ***Normal hearing / hearing loss/ children*** / ***adults*** / elderly / veterans / ***low-resource environment*** / ototoxic- / self- / noise- monitoring / infectious disease |
| **Efficiency** | ***Testing time*** / number of stimuli / not reported  mean testing time 5 minutes for bilateral air conduction audiogram in normal hearing and hearing impaired [42] |

1. Van Tassel & Folkeard

| **Introduction:** tablet-based self-measurement of auditory thresholds  **Selected reports:** Van Tasell & Folkeard, 2013 [83]  **Additional reports:** none | |
| --- | --- |
| **Threshold Seeking Method and Range** | |
| **Frequencies** | ***clinical*** / high-resolution /reduced / extended range /not reported |
| **Intensities** | ***clinical*** / extended / reduced / not reported |
| **Masking** | automated /manual / other / ***no*** / not reported |
| **Seeking approach** | ***(modified) Hughson-Westlake*** / MLAG / Bekesy tracking / ***other*** |
| **Response Method and Presentation of Results** | |
| **Response method** | forced choice / ***single response*** / not reported |
| **Test paradigm** | ***self-test*** / facilitated by operator |
| **Presentation of results** | ***conventional*** / high-resolution representation / automated classification / not reported |
| **Test Equipment** | |
| **Transducers** | ***air conduction***/ bone conduction  (ER-3A) |
| **Calibration** | ***conventional*** / unconventional / no / not reported |
| **Hardware** | portable audiometer / computer-based / web-based / smartphone / ***tablet*** |
| **Test Quality Control** | |
| **Comparison automated versus manual** | ***RMSD*** < 10 dB / ***< 6 dB*** / statistical equivalence / statistically not equivalent / not reported |
| **Test-retest** | ***RMSD*** < 10 dB / ***< 6 dB*** / statistical equivalence / statistically not equivalent / not reported |
| **Deals with** | false-responses / noise control |
| **Validation Approach, Test Population, and Context** | |
| **Validation** | ***gold standard*** / reasonable standard / proof of concept  55 subjects included, 22-86 years |
| **Test Population** | ***Normal hearing /*** ***hearing loss*** / children / ***adults*** / elderly / veterans / low-resource environment / ototoxic- / self- / noise- monitoring / infectious disease |
| **Efficiency** | Testing time / number of stimuli / ***not reported*** |

1. Vinay et al, NEWT

| **Introduction:** A new automated method termed as 'New Early Warning Test (NEWT)' has been designed to provide automatic auditory threshold monitoring in individuals exposed to high noise levels. The NEWT method is incorporated inside an active communication earplug called Quietpro®, which has high attenuation characteristics for background noise.  **Selected reports:** Vinay et al., 2014 [38,49]  **Additional reports:** none | |
| --- | --- |
| **Threshold Seeking Method and Range** | |
| **Frequencies** | clinical / high-resolution /***reduced*** / extended range /not reported |
| **Intensities** | clinical / extended / ***reduced*** / not reported |
| **Masking** | automated /manual / other / ***no*** / not reported |
| **Seeking approach** | (modified) Hughson-Westlake / MLAG / Bekesy tracking / ***other*** |
| **Response Method and Presentation of Results** | |
| **Response method** | ***forced choice*** / single response / not reported |
| **Test paradigm** | ***self-test*** / facilitated by operator |
| **Presentation of results** | ***conventional*** / high-resolution representation / automated classification / not reported |
| **Test Equipment** | |
| **Transducers** | ***air conduction***/ bone conduction  (Quietpro HDP unit) |
| **Calibration** | ***conventional*** / unconventional / no / not reported |
| **Hardware** | portable audiometer / ***computer-based*** / web-based / smartphone/tablet |
| **Test Quality Control** | |
| **Comparison automated versus manual** | RMSD < 10 dB / < 6 dB / ***statistical equivalence*** / statistically not equivalent / not reported |
| **Test-retest** | RMSD < 10 dB / < 6 dB / ***statistical equivalence*** / statistically not equivalent / not reported |
| **Deals with** | false-responses / ***noise control*** |
| **Validation Approach, Test Population, and Context** | |
| **Validation** | ***gold standard*** / reasonable standard / proof of concept  22 subjects included, 25-37 years [38],  16 subjects included, 24-62 years [49] |
| **Test Population** | ***Normal hearing*** / hearing loss / children / **adults** / elderly / veterans / low-resource environment / ototoxic- / self- / ***noise- monitoring*** / infectious disease |
| **Efficiency** | ***Testing time*** / number of stimuli / not reported  Mean testing time 1.5 minutes, for automated unilateral partial audiogram (1, 3, 4 and 6 kHz) air conduction audiogram in normal hearing (3 minutes manually) [49]. |

1. Whitton et al

| **Introduction:** a self-administered audiometric software application which can be used at home  **Selected reports:** Whitton et al., 2016 [84]  **Additional reports:** none | |
| --- | --- |
| **Threshold Seeking Method and Range** | |
| **Frequencies** | clinical / high-resolution /reduced / ***extended range*** /not reported |
| **Intensities** | ***clinical*** / extended / reduced / not reported |
| **Masking** | ***automated*** /manual / other / no / not reported |
| **Seeking approach** | ***(modified) Hughson-Westlake*** / MLAG / Bekesy tracking / other |
| **Response Method and Presentation of Results** | |
| **Response method** | forced choice / ***single response*** / not reported |
| **Test paradigm** | ***self-test*** / facilitated by operator |
| **Presentation of results** | ***conventional*** / high-resolution representation / automated classification / not reported |
| **Test Equipment** | |
| **Transducers** | ***air conduction***/ bone conduction  (Bose AE2i consumer-grade circumaural) |
| **Calibration** | ***conventional*** / unconventional / no / not reported |
| **Hardware** | portable audiometer / computer-based / web-based / smartphone/ ***tablet*** |
| **Test Quality Control** | |
| **Comparison automated versus manual** | RMSD < 10 dB / < 6 dB / ***statistical equivalence*** / statistically not equivalent / not reported |
| **Test-retest** | RMSD < 10 dB / < 6 dB ***/ statistical equivalence*** / statistically not equivalent / not reported |
| **Deals with** | false-responses / ***noise control*** |
| **Validation Approach, Test Population, and Context** | |
| **Validation** | gold standard / ***reasonable standard*** / proof of concept  19 subjects included, 25-82 years |
| **Test Population** | ***Normal hearing / hearing loss*** / children / ***adults*** / elderly / veterans / low-resource environment / ototoxic- / self- / noise- monitoring / infectious disease |
| **Efficiency** | Testing time / number of stimuli / ***not reported*** |

1. Yeung et al, Shoebox

| **Introduction:** SHOEBOX developed an iPad audiometer. For more information: https://www.shoebox.md/  **Selected reports:** Yeung et al., 2013 [44,56,85–89]  **Additional reports:** none | |
| --- | --- |
| **Threshold Seeking Method and Range** | |
| **Frequencies** | clinical / high-resolution /reduced / ***extended range*** /not reported |
| **Intensities** | clinical / extended / ***reduced*** / not reported |
| **Masking** | ***automated*** /manual / other / no / not reported |
| **Seeking approach** | ***(modified) Hughson-Westlake*** / MLAG / Bekesy tracking / other |
| **Response Method and Presentation of Results** | |
| **Response method** | ***forced choice / single response*** / not reported |
| **Test paradigm** | ***self-test / facilitated by operator*** |
| **Presentation of results** | ***conventional*** / high-resolution representation / automated classification / not reported |
| **Test Equipment** | |
| **Transducers** | ***air conduction/ bone conduction***  (TDH-50, ER-3A inserts and speakers) |
| **Calibration** | ***conventional*** / unconventional / no / not reported |
| **Hardware** | portable audiometer / computer-based / web-based / smartphone/ ***tablet*** |
| **Test Quality Control** | |
| **Comparison automated versus manual** | ***RMSD < 10 dB*** / < 6 dB / statistical equivalence / statistically not equivalent / not reported |
| **Test-retest** | ***RMSD < 10 dB*** / < 6 dB / statistical equivalence / statistically not equivalent / not reported |
| **Deals with** | false-responses / ***noise control*** |
| **Validation Approach, Test Population, and Context** | |
| **Validation** | ***gold standard***  70 subjects included, 3-13 years [85],  33 subjects included, 18-65 years [56],  40 subjects included, 19-87 years [87],  49 subjects included, 4-88 years [86],  70 subjects included, 5-52 years [89];  126 subjects included, 24-42 years [88]  ***reasonable standard*** / proof of concept  79 subjects included, 5-17 years [44] |
| **Test Population** | ***normal hearing / hearing loss / children / adults*** / elderly / veterans / ***low-resource environment*** / ***ototoxic-*** / self- / noise- ***monitoring*** / infectious disease |
| **Efficiency** | ***Testing time*** / number of stimuli / not reported  Mean testing time 2 minutes for automated bilateral air conduction audiogram in normal hearing children. Mean testing time 5 minutes for automated bilateral air conduction audiogram in hearing impaired children [85]  mean testing time 10-15 minutes for automated bilateral air conduction (including extended frequencies) audiogram in normal hearing and hearing impaired adults [88]. |
