# Supplementary material for: Digital Approaches to Automated and Machine Learning Assessments of Hearing: Scoping Review
Source: J Med Internet Res. 2022 Feb 2;24(2):e32581. doi: 10.2196/32581 (PMC8851345; doi:10.2196/32581)
Supplement: Multimedia Appendix 5 [file jmir_v24i2e32581_app5.docx]

**Supplementary Material Table 5: Types of Statistical Analyses for Accuracy and Reliability**

| **Type of analysis** | **Number of approach-clusters** | **References** |
| --- | --- | --- |
| ***Accuracy (threshold comparison with manual audiometry)*** | |  |
| Average differences and standard deviation | **10** | [35,37,39,45,49,58,65,67,81,84] |
| Average thresholds and standard deviation | **1** | [32] |
| RMSD | **14** | [23,36,40,43,48,50,55,59,68,70,74,77,83,85] |
| Linear regression and correlation coefficients | **1** | [33] |
| ANOVA analysis | **1** | [34] |
|  |  |  |
| ***Test-retest reliability*** | |  |
| Average differences and standard deviation | **6** | [39,49,65,67,83,84] |
| RMSD | **7** | [23,48,50,59,68,74,77,85] |
| Pearson Product moment correlation coefficients | **2** | [35,36] |
| Standard of variance | **1** | [37] |
| Repeated ANOVA | **1** | [344] |
